# Integrated 16S rRNA sequencing and metabolomics analysis to investigate the antidepressant role of Yang-Xin-Jie-Yu decoction on microbe-gut-metabolite in chronic unpredictable mild stress-induced depression rat model

**DOI:** 10.3389/fphar.2022.972351

**Published:** 2022-09-30

**Authors:** Xing-Qiu Liang, Peng-Yu Mai, Hui Qin, Sen Li, Wen-Juan Ou, Jian Liang, Jing Zhong, Ming-Kun Liang

**Affiliations:** ^1^ Medical College, Guangxi University, Nanning, China; ^2^ Department of Science and Technology, Ruikang Hospital Affiliated to Guangxi University of Chinese Medicine, Nanning, China; ^3^ Guangxi International Zhuang Medicine Hospital, Nanning, China; ^4^ School of Basic Medical Sciences, Guangxi University of Chinese Medicine, Nanning, China

**Keywords:** gut microbiota, metabolism, Traditional Chinese Medicine, adolescent depression, microbiota-gut-brain axis

## Abstract

**Objectives:** Our goals were to evaluate the antidepressant efficacy of Yang-Xin-Jie-Yu Decoction (YXJYD) in Chronic Unpredictable Mild Stress (CUMS)-induced depression rat model and to investigate the underlying mechanisms.

**Design:** We used CUMS-induced depression rat model to evaluate whether oral administration of YXJYD with different doses (2.1 g/kg, 1.05 g/kg and 0.525 g/kg, respectively) improve the depressive-like symptoms, and then performed UHPLC-Q-TOF-MS to explore the active ingredients of YXJYD. Subsequently, rat’s cecal contents, serum, and urine were collected from the control group, CUMS model group, and YXJYD high-dose (2.1 g/kg) treatment group. The 16S rRNA sequencing was performed on the cecal contents, based on Illumina MiSeq platform, and ANOVA analysis were used to analyze the composition variety and screen differential expression of gut bacteria in the three groups. ^1^H Nuclear Magnetic Resonance (NMR) analysis was used for analyzing the metabolites obtained from cecal contents, serum, and urine, and KEGG enrichment analysis was used to identify pathways of differential metabolites. An integrated 16S rRNA sequencing and metabolomic data were conducted to characterize the underlying mechanisms of YXJYD

**Results:** The gut microbial communities, and serum, cecal content, urine metabolic compositions were significantly significantly altered in CUMS-induced depressive rats, while YXJYD effectively ameliorated the CUMS-associated gut microbiota dysbiosis, especially of *Monoglobus,* and alleviated the disturbance of serum, cecal content, urine metabolome and reversed the changes of key depressive and gut microbiota-related metabolites, such as succinic acid, taurine, hippuric acid, melatonin. With an integrated study of the gut microbiota and metabolomes, we identified the pathway of tricarboxylic acid cycle (TCA cycle) and propanoate metabolism as the regulated target of YXJYD on host-microbiome interaction.

**Conclusion:** Our findings further confirmed the imbalance of metabolism and intestinal microbial is closely related to CUMS-induced depression. YXJYD regulates gut microbiome to affect body metabolomes and then produce antidepressant-like effect in CUMS-induced depressive rats while its molecular mechanism possibly be increased *Monoglobus* abundance in gut microbiota and regulated the TCA cycle pathway and propanoate metabolism in host.

## 1 Introduction

The prevalence of adolescent depression increased at a faster pace than adult depression due to it is unique period of physical development along with severe lifestyle changes (Mojtabai, Olfson, and Han 2016; Twenge et al., 2018; Simkin 2019). It has been observed a strong link between depression and suicide, and that finally lead to a ninefold rise in the incidence of suicidal thoughts among teenagers, particularly in females (Lara et al., 2018; Simkin 2019). The above grim situation highlighted the limits of available treatments. According to the data reported by several meta-analyses, most antidepressants do not provide obvious benefits above placebo for adolescents (Cipriani et al., 2016; Ignaszewski and Bruce, 2018). Moreover, another comprehensive meta-analysis indicated that the efficacy of medications for children and adolescents with MDD were far from satisfactory and also indicated that venlafaxine might increase the risk for suicidality (suicidal behavior or ideation) in young people (Cipriani et al., 2016). What’s more, depression in adolescence predicts depression and anxiety in adulthood, and most of the affected adults had their first depressive episode during adolescence (Simkin 2019). Worsely, due to the particularity of physical development, the side effects of antidepressant drug, such as headache, stomach discomfort, drowsiness or sleeplessness, and dry mouth, are more prevalent in adolescence than in adults. This lead to the antidepressants choice for youth is highly restricted and also might eventually bring about drug withdrawal. Given all, there is an urgent need to find effective antidepressants but without adverse effects for teenage depression.

Adolescent depression is not a unified syndrome, in which multiple underlying mechanism also coexist. Thus, the best therapy strategy is to identify the unique cause for each individual patient and then employ a tailored treatment to address not just depression, but also the body’s malfunction that causes depressive symptoms (Zhang and Cheng 2019; Li et al., 2020). Growing evidence has demonstrated that the gut microbiota disruption is closely associated with a variety of mental diseases, which mainly showed in changes of gut microbiota diversity and composition (P. Zheng et al., 2016; Yang et al., 2020). Comparing with healthy people, the phylum of *Bacteroidetes, Proteobacteria, Firmicutes* showed a higher proportion in major depressive disorder (MDD), while the phylum of *Actinobacteria* showed a lower. Moreover, the abundance of the genus *Bacteroides* was increased in MDD patients, while that of the genera *Blautia* and *Eubacterium* were decreased (Yang et al., 2020). The specific features of gut microbiota in different populations distinguish adolescents from adults. Thus, targeting on the gut microbiota may provide a manageable and personalized therapy for adolescent depression.

Up until now, Traditional Chinese Medicine (TCM) has been utilized to treat depression. Its holistic, multidrug, and multitarget character aligns well with the therapeutic concept of systemic medicine in the treatment of teenage depression. Yang-Xin-Jie-Yu decoction (YXJYD) is an empirical botanical drug prescription for adolescent depression made up of ten commonly used plants: *Panax ginseng* C.A.Mey., *Ophiopogon japonicus* (Thunb.) Ker Gawl*.*, *Schisandra chinensis* (Turcz.) Baill., *Epimedium sagittatum* (Siebold & Zucc.) Maxim., *Allium chinense* G.Don, *Rosa rugosa* Thunb., *Albizia julibrissin* Durazz., *Curcuma aromatica* Salisb., *Acorus calamus L.*, and *Citrus × aurantium* L. It should be noted that *Panax ginseng* C.A.Mey., *Ophiopogon japonicus* (Thunb.) Ker Gawl., and *Schisandra chinensis* (Turcz.) Baill. are compositions of Sheng Mai Yin (SMY) prescription, which has traditionally been used to protect the heart from a variety of cardiac illnesses. In both humans and rats, modern pharmacology discovered that SMY might enhance coronary blood flow, improve myocardial tolerance to hypoxia, and act as a stimulant similar to interrenalin (Z. Chen et al., 2018). Besides, the plants of *Epimedium sagittatum* (Siebold & Zucc.) Maxim. (Wu et al., 2013; Liu et al., 2015), *Allium chinense* G.Don (Yin et al., 2019), *Rosa rugosa* Thunb. (Fekadu, Shibeshi, and Engidawork 2016; Mingkun et al., 2021), *Albizia julibrissin* Durazz. (Chunlei, Yongquan, and Xueli 2012; Xueli et al., 2013), *Curcuma aromatica* Salisb. (Haibing, Yi, and Guojun 2012), *Acorus calamus L.* (Tengfei et al., 2012), and *Citrus × aurantium* L. (Li et al., 2013; Chengfu et al., 2014) are commonly used for antidepressant single or complex by multiple anti-depression mechanism. *Acorus calamus L.* was also shown to have an antidepressant effect on CUMS rats via modulating the expression of monoamine neurotransmitters and their receptors (Tengfei et al., 2012), while *Curcuma aromatica* Salisb. alleviated the depressive symptoms by affecting on the neuroplasticity of hippocampus neurons (Haibing, Yi, and Guojun 2012).

It is generally recognized that a TCM botanical drug formula is more effective than single botanical drug molecules or botanical drugs in clinical practice, and YXJYD shown notable antidepressant effect on teenagers with depression when compared to any other single botanical drugs in clinical practice (Hsiao and Liang, 2010). Moreover, patient comments revealed that YXJYD boosted their appetite and alleviated several digestive problems. We postulated that YXJYD’s antidepressant impact was related to repair gut microbiota dysbiosis and subsequently regulating the body’s metabolism. To test this hypothesis, we explored the effect of YXJYD on cecal content, urine, and serum metabolites in CUMS-induced depression rat model by untarget metabolomics, and then investigated the effect of YXJYD on cecal microbiome in CUMS-induced depression rats by 16S rRNA sequencing. We effectively demonstrated that the antidepressant mechanism of YXJYD was intimately related with the gut microbiota and body’s metabolism, and that YXJYD had holistic, multidrug, and multitarget qualities that make it a potential medication for depression.

## 2 Materials and methods

### 2.1 Experimental reagents

Fluoxetine hydrochloride (Patheon France, France, production batch number: 21201A) was provided by Ruikang hospital affiliated to guangxi university of Chinese medicine. The ELISA assay kit for cortisol (202112) was purchased from the Shanghai Enzyme-linked Biotechnology Co., Ltd. (Shanghai, China). QlAamp DNA Stool Mini Kit (50) was purchased from Shanghai Canspec Scientific Instruments Co., Ltd. (catalog [cat.] no. 51604; Qiagen, Germany). Qubit dsDNA HS ASSAY Kit was obtained from Invitrogen and Life technologies company (CA, United States). DL2000 DNA Maker was obtained from Takara Bio INC. (Kyoto, Japan). The 50×TAE buffer was obtained from Shanghai Shenggong Bioengineering Co., Ltd. (Shanghai, China). UPLC-Q-TOF/MS grade acetonitrile and HPLC grade acetonitrile, formic acid, were provided by Merck & Co., Inc. (Merck, Germany). Animals Male Sprague-Dawley (SD) rats were obtained from the Laboratory Animal Center of Guangxi Medical University (license number: SCXK Gui 2020-0003). The rats were maintained at the Guangxi Medical University Animal Center with a 12 h light-dark cycle and fed for 1 week before to the experiment to minimize the stress reaction (22 ± 3°C with a relatively constant humidity of 55 ± 15%). Except during the study period, rats were fed and water freely. All animal experiments were in accordance with the Guide for the Care and Use of Laboratory Animals approved by the Institutional Animal Care and Use Committee at Guangxi Medical University (license number: SYXK Gui 2020-0004).

### 2.2 Drug preparation

Yang Xin Jie Yu decoction is composed as follows: *Panax ginseng* C.A.Mey. (15 g, Batch number: 210901), *Ophiopogon japonicus* (Thunb.) Ker Gawl*.* (15 g, Batch number: 210901), *Schisandra chinensis* (Turcz.) Baill. (15 g, Batch number: 210501), *Epimedium sagittatum* (Siebold & Zucc.) Maxim. (15 g, Batch number: 210501), *Allium chinense* G.Don (15 g, Batch number: 210501), *Rosa rugosa* Thunb. (15 g, Batch number: 210801), *Albizia julibrissin* Durazz. (He Huan Hua), *Curcuma aromatica* Salisb. (15 g, Batch number: 210901), *Acorus calamus L.* (15 g, Batch number: 210901), and *Citrus × aurantium* L (10 g, Batch number: 210901). (Details are showed in Table 1). All medicinal materials were provided by the Ruikang hospital affiliated to Guangxi university of Chinese medicine. The certificate specimens of each botanical drugs were identified by the pharmacy, and quality inspection reports were provided. All medicinal materials were soaked with water for 30 min at a solid liquid ratio of 1:8 (g/ml), and were then extracted three times under reflux for 2 h. All the supernates were mixed, and the solvents were then removed by using a rotary evaporator at 60°C to obtain YXJYD extracts, and subsequently freeze-dried and smashed into granules (total weight 31.30 g) for experimental use. The lyophilized YXJYD granule was thoroughly dissolved in 100 ml distilled water to make a solution containing 1.45 g raw drugs /mL for the future study.

**TABLE 1:** Details of the herbal pieces in YXJYD.

Botanical plant name	Family and plant part used	English name	Chinese name	Dry weight of crude drugs in YXJYD (g)
*Panax trifolius L.*	*Araliaceae*, root	*Ginseng*	Ren-shen	15
*Ophiopogon japonicus (Thunb.) Ker Gawl.*	*Liliaceae*, root	*Ophiopogonis Radix*	Mai-dong	15
*Schisandra chinensis (Turcz.) Baill.*	*Schisandraceae*, fruit	Schisandrae Chinensis Fructus	Wu-wei-zi	15
*Epimedium rotundatum K.S.Hao*	*Berberidaceae*, Leaves basal and cauline	Epimedii Folium	Yin Yang Huo	15
*Allium macrostemon Bunge*	*Liliaceae*, root	Bulbus Allii Macrostemonis	Xie Bai	15
*Rosa Abyssinica Lindley (Rosaceae)*	*Rosaceae*, flower	Flos Rosae Rugosae	Mei Gui Hua	15
*Acacia julibrissin (Durazz.) Willd*	*Leguminosae, flower*	Flos Albiziae	He Huan Hua	15
*Curcuma aeruginosa Roxb*	*Zingiberaceae*, root	Radix Curcumae	Yu Jin	15
*Acori Tatarinowii Rhizoma*	*Araceae*, root	Acori Tatarinowii Rhizoma	Shi Chang Pu	15
*Pericarpium Citri Reticulatae Viride*	*Rutaceae*, fruit	Green Tangerine Peel	Qing Pi	10

### 2.3 The component analysis of YXJYD

The solution of YXJYD granules was filtered out by 0.22 µm membrane-filter and injected into an Ultra-High Performance Liquid Chromatography analytical system (Waters Corp., Milford, MA, United States) with ACQUITY UPLC HSS T3 C18 column (100mm×2.1 mm, 1.8 μm i.d., Waters, Unites States) at the column temperature: 30°C. The mobile phases were 1% formic acid in water (A) and acetonitrile (B), and the elution conditions were: 0–0.5 min: 98%A; 0.5–5.0 min: 98%-70%A; 5.0–15.0 min: 70%-60%A; 15.0–20.0 min: 60%-40%A; 20.0–21.0 min: 40%-15%A; 21.0–23.0 min: 15%-60%A; 24.0–25.0 min: 60%-98%A.

Mass data was obtained from a XEVO-G2-S-QTOF-MS mass spectrometer equipped with an electrospray ionization source (ESI) (Waters Corp., Massachusetts, United States). The MS detection parameters were set as follows: collision voltage (capillary voltage): 3.0 kV (negative ion: 1.8 kV); sample and extraction cone voltage: 40 V (negative ion: 40 V) and 4.0 V; desolvation gas rate and temperature (negative ion: 4.0 V): 600 L/h and 350°C (negative ions: 150°C); cone gas rate: 50 L/h; ion source temperature: 100°C (negative ions: 100°C); scan time: 0.2 s; scan interval delay (interscan delay): 0.02 s. The leucine-enkephalin (mass-to-charge ratio in positive ion mode: 556.2771, mass-to-charge ratio in negative ion mode: 554.2615) was selected as the real-time calibration substance, and its concentration and flow rate were 500 ng/ml and 10 μL/min, respectively. The mass-to-charge ratios of the primary mass spectrometry data ranged from 100 to 1,500, whereas the secondary mass spectrometry data ranged from 50 to 1,500 Hz.

### 2.4 Drug administration

**FIGURE 1:**
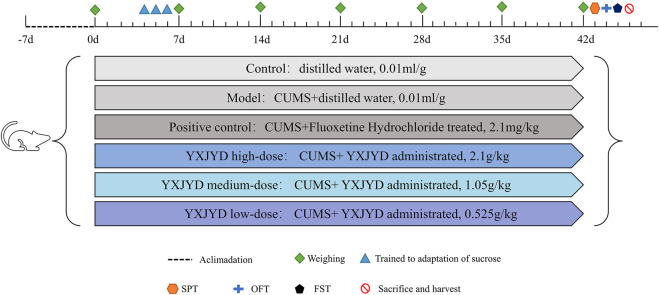
Animal experimental designed.

After 1 week of acclimatization, 90 SD rats (6 weeks old) were randomly divided into six groups: control group (n = 15, distilled water administrated, 0.01 ml/g), model group (n = 15, distilled water administrated, 0.01 ml/g), positive control group (n = 15, Fluoxetine Hydrochloride treated, 2.1 mg/kg), YXJYD high-, medium-, and low-dose group (*n* = 15 per group, YXJYD administrated, 2.1 g/kg, 1.05 g/kg and 0.525 g/kg, respectively). These dosages were calculated from the clinical dosages for adolescents and the equivalent conversion of the body surface area between animals and humans. The experimental design is showed in Figure 1. Drug administration was performed a week before CUMS.

### 2.5 Chronic unpredictable mild stress

The rats were placed individually and subjected to mild stress for 5 weeks except the control group. The stresses were as follows: 45 cage tilt for 24 h from 10 to 10 am in the next day, bound for 3 h, water deficiency for 24 h, fasted for 24 h, 200 r/min shaking for 15 min, tail-clamping for 5 min, wet bedding for 24 h (200 ml water/100 g bedding), swimming in cold water for 5 min (the temperature was below 10°C), light and shape reversed, lightening throughout the night (100 W light for 36 h), and so on.

### 2.6 Behavioral tests

#### 2.6.1 Sucrose preference test

72 h before the test, rat were trained to adapt to 1% sucrose solution (W/V). Two bottles were placed in each cage, both were 1% sucrose solution for 24 h, next one bottle was 1% sucrose solution and another was water for 24 h, and the last 24 h were deprived of water and food. During the test, rats were housed in individual cages and had free access to two bottles with sucrose solution (100 ml, 1%, W/V) and tap water (100 ml), respectively. Twelve hours later, sucrose and water consumption (ml) were recorded, and the sucrose preference was calculated as following:

$$sucrose\;preference\left(\%\right)=\frac{\;sucrose\;consumption\;volume\;\left(ml\right)}{sucrose\;consumption\;volume\;\left(ml\right)+water\;consumption\;volume\;\left(ml\right)}\times 100\%$$

#### 2.6.2 Open field test

The rats were left alone in a self-made PC box (50 cm × 50 cm × 50 cm) with the arena’s floor split into 100 equal squares (5 cm × 5 cm) and allowed rats explore freely. Each rat was placed individually in the center of the arena in a calm room, allowing for 5 min of unfettered exploration. A camera was placed above the open box to record locomotor activity during the experiment. A 5-min test was used to determine the overall distance traveled and time spent in the center region. Before using another animal, the test apparatus was cleaned completely with 75% ethanol.

#### 2.6.3 Forced swimming test

The experiment was carried out in a self-made cylindrical container (50 cm height; 30 cm in diameter) filled to a depth of 40 cm with water (at a temperature of 24°C). During the last 5 min of the experiment, the rat’s immobility was assessed. Immobility was defined as having the limbs fixed or the forelimbs floating slightly (but the body remained immobile). Immobility was defined as their passive floating in the water during the test. Replacement of clean water before introducing new rats to avoid tainting the findings due to a waste water quality issue created by the prior.

### 2.7 Blood sampling collection

Blood was collected after behavior test by ntraperitoneal injection with 20% urethane to anaesthetize the rat. Blood was collected in rat aortic blood immediately, and then sampled into 5 ml blood collection tubes. After standing in the room temperature for 2 h, the serum was obtained following a 10 min centrifugation at 3,000 g (Hermle Z 300 K) at 4 °C, and stored at -80°C.

### 2.8 Determination of cortisol levels in the serum by ELISA

The determination of cortisol level were performed by using a commercial enzyme immunoassay kit (Shanghai Enzyme-linked Biotechnology Co., Ltd.) according to the manufacturer’s instructions. All samples were tested in duplicate.

### 2.9 Urine, serum, and cecum contents collection

The night before sacrificed, rats’ urine was collected for 24 h, and cecum contents was taken from the cecum after they were sacrificed. Blood samples were collected after the behavioral test. The rats were anesthetized and blood was immediately collected from the abdominal aorta in 5 ml of vacuum blood collection tubes without anticoagulant, left at room temperature for 2 h, centrifuged at 4°C for 10 min at 3,000 rpm, and the supernatant was collected in new centrifuge tubes. After the rats were sacrificed, the contents of the cecum were collected. All samples were stored in the refrigerator at -80 °C.

### 2.10 ^1^H NMR analysis for metabolomics

#### 2.10.1 Preparation of urine, serum, and cecum content for ^1^H NMR analysis

Cecum content samples: 100 mg cecum content samples were suspended in 1 ml PBS (0.1 mol /L, pH 7.4). The samples were homogenated in an ice bath for 2 times, 15 s at a time, and then stood for 10 s. After ultrasonicating for 30 min, centrifuging to remove deposition (14000 g, 15 min, 4°C). Afterward, 450 μL of the supernatant was added with 50 μl of D_2_O containing 0.0025% (mass to volume ratio) TMSP. After being mixed by vortexing for 1 min, the mixture was transferred into a 5 mm NMR tube.

Urine sample: 500 μl urine sample were taken to 2 ml EP tube. After centrifuging to remove deposition (3,500 rpm, 15 min, 4°C), 450 μl of the supernatant were added in 50 μl PBS (0.1 mol /L, pH 7.4), and then vortexed for 1 min, centrifuging to remove deposition (12000 rpm, 15 min, 4°C). Afterward, 450 μL of the supernatant was added with 50 μl of D_2_O containing 0.0025% (mass to volume ratio) TMSP. After being mixed by vortexing for 1 min, the mixture was transferred into a 5 mm NMR tube.

Serum sample: After thawing, 250 μl serum sample were taken in 2 ml EP tube, and 250 μl PBS (0.1 mol /L, pH 7.4) were added. After vortexing for 1 min, the mixture were centrifuged to remove deposition (14000 g, 15 min, 4°C). Afterward, 450 μl of the supernatant was added with 50 μl of D2O containing 0.0025% (mass to volume ratio) TMSP. After being mixed by vortexing for 1 min, the mixture was transferred into a 5 mm NMR tube.

#### 2.10.2 ^1^H NMR measurement

^1^H NMR was performed on the Bruker 600 MHz AVANCE III NMR instrument (Karlsruhe, Germany) at the operating temperature of 25°C. The ^1^D CPMGpr1d Bruker standard sequence was applied for feacal sample and serum sample to scan 64 times, while the NOSEY gppr1d Bruker standard sequence was for urine sample to scan 32 times. Fixed receiver gain, the detection spectrum width was 0.3 Hz, the pulse width was 20 ppm, and the relaxation delay was 5 s.

#### 2.10.3 ^1^H NMR data analysis

The raw ^1^H NMR data were adjusted using MestReNova 11.0 (Santiago, Spain) to set TSP peaks at 0 ppm, shear water peaks (stool (4.24–6.00 ppm), urine (4.28–6.00 ppm), serum (4.12–7.08 ppm), baseline and peak extraction, and peak integration automation. To reduce baseline noise being identified as variable information, total area normalization was subsequently performed and peak area cut-offs that were performed at 0.04 ppm intervals. The processed data exported as .txt format files, while the identified fractions were used as variables for the differential substances to be screened, edited in a fixed format and imported into SIMCA-P 14.1 software for multivariate statistical analysis, including principal component analysis (PCA), partial least squares-discriminant analysis (PLS-DA) and orthogonal partial least squares-discriminant analysis (OPLS-DA). For the OPLS-DA mathematical model developed for potential biomarker screening, after 200 permutation tests to confirm that the model was not overfitted, the results were used for potential biomarker screening with the absolute value of Variable importance on projection (VIP) > 1 and *p* < 0.05. The peaks at different ppm were identified using Chenomx NMR suite 8.6 software, and the identification results were verified using ^1^H-^1^H coupling on the compounds and 2D NMR spectra with ^1^H-^1^H COSY coupling information.

### 2.11 Analysis of gut microbiota by 16S rRNA sequencing

Cecal contents were collected from the cecum, and total DNA was extracted from stool samples using the QIAmp DNA microbiome kit (Qiagen, German) according to the manufacturer’s protocol. After determining the DNA concentration and integrity, an amplicon sequencing library was constructed based on the PCR-amplified V3–V4 variable region of 16S rRNA. Then, qualified libraries was used on the Illumina MiSeq platform for paired-end sequencing according to the manufacturer’s instructions. The software of Trimmomatic, FLASH, and QIIME software were used to filter the original sequencing data. UPARSE software was used to cluster the clean readings into operational taxonomic units (OTUs) with the threshold of 97%. Then, QIIME package was used to select the representative reads from each OUT, and RDP Classifier software v.2.13 was used to annotate and classify representative OTU sequences against the SILVA 138 16S rRNA database using a confidence threshold of 70%. Alpha diversity and beta diversity analysis were performed by R software (version 3.3.1). Bray-Curtis dissimilarity matrix was developed with the normalized sequences. Hierarchical cluster analysis (HCA), principal coordinates analysis (PCoA), and distance between two groups were performed by the Bray-Curtis dissimilarity matrix. The linear discriminant analysis (LDA) effect size (LEfSe) method was applied to reveal the effect of each differentially abundant taxon and distinguish the key phenotypes responding to the YXJYD treatment, with a set logarithmic LDA score of 2.0. Functional classification schemes of KEGG (Kyoto Encyclopedia of Genes and Genomes) Orthology were predicted by Tax4Fun.

### 2.12 Statistical analysis

The results of behavioral tests and cortisol levels determination were compared among using one-way ANOVA by GraphPad Prism (9.0.0). Pairwise comparison of microbiome composition was conducted using Metastats based on the Majorbio Cloud Platform (https://cloud.majorbio.com/page/tools/). The Spearman correlation coefficient was used to analyze the correlations. *p* < 0.05 or FDR < 0.05 was considered statistically significant. For alpha diversity, the Wilcoxon rank-sum test *p* < 0.05 was considered statistically significant. For beta diversity, distance of PcoA between two groups were performed based on the Bray-Curtis dissimilarity matrix and ANOSIM test (permutations = 999, *p* < 0.001). The linear discriminant analysis (LDA) efect size (LEfSe, https://huttenhower. sph.harvard.edu/galaxy/) was used to identify biomarker taxa responsible for discrimination. Random forest (RF) analysis was performed by the R package “randomForest”, and the mean decrease accuracy (MDA) score was used to determine the importance ranking of key taxa for classifcation (Statnikov et al., 2013; Miao et al., 2022).

## 3 Results

### 3.1 The chemical constituents of YXJYD

**FIGURE 2:**
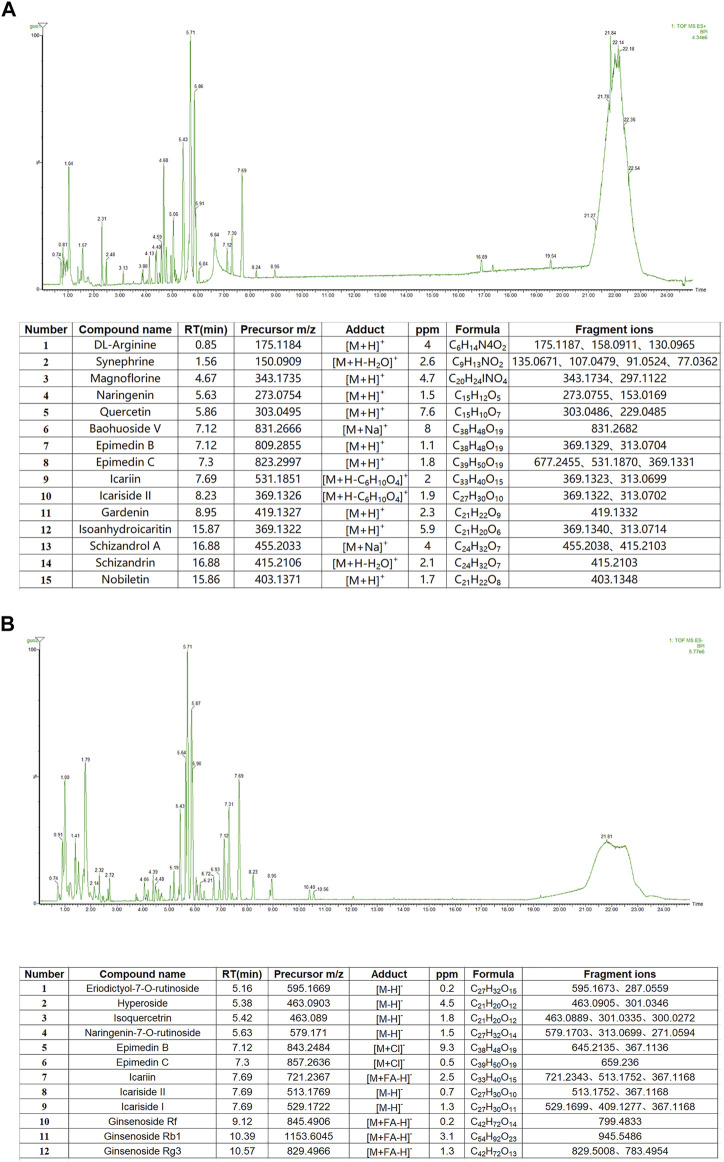
**(A)** Base peak chromatograms and recognized main compounds of YXJYD extract in ESI (+) mode. **(B)** Base peak chromatograms and recognized main compounds of YXJYD extract in ESI (−) mode.

UPLC-Q-TOF/MS was used to characterize the chemical composition of YXJYD in positive and negative ion modes, and the characteristic chromatogram and results of YXJYD were shown in Figure 2 and Supplementary Figures S2–S3. There were fifteen chemicals discovered in the positive ion mode and twelve compounds detected in the negative ion mode.

### 3.2 Exposure to chronic unpredictable mild stress is sufficient to induce depressive-like behaviors

**FIGURE 3:**
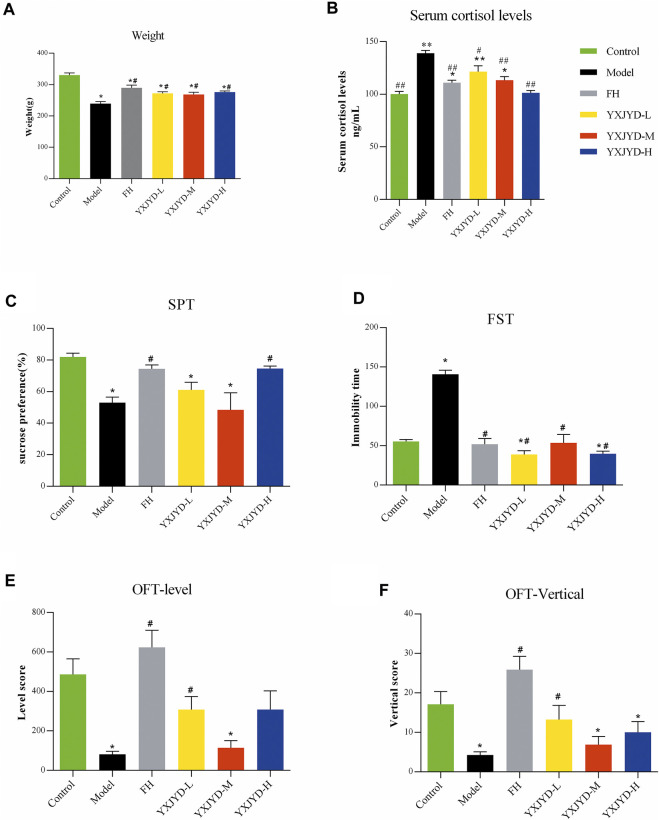
The effect of YXJYD administration on CUMS-induced depressive behaviors. Behavioral tests results. **(A)** Effect of YXJYD on the rats’ weight; **(B)** Effect of YXJYD on the rats’ serum cortisol levels; **(C)** the sugar preference test after YXJYD administration; **(D)** the forced swimming test after YXJYD administration; **(E)** the level score of open field test after YXJYD administration; **(F)** the vertical score of open field test after YXJYD administration. vs. control group, *p < 0.05, **p < 0.01, ***p < 0.001; vs. model group, ^#^ *p* < 0.05, ^##^ *p* < 0.01; ^###^ *p* < 0.001. (Rat for behavioral tests, n; Control = 14, Model = 14, FH = 13, YXJYD-L = 12, YXJYD-M = 8, YXJYD-L = 13). YXJYD-L, low-dose administration of YXJYD; YXJYD-M, medium-dose administration of YXJYD; YXJYD-H, high-dose administration of YXJYD; FH, Fluoxetine Hydrochloride treated; SPT, sugar preference test; FST, forced swimming test; OFT, open field test.

To create an adolescent depressive rat model, we employed chronic unpredictable mild stress to stimulate the rats (6 weeks old) to induce depressive-like behaviors. In comparison with control group, CUMS model rats displayed expected behavioral alterations, such as reduced exploratory activity in OFT (*p* < 0.05), decreased sucrose preference in SPT (*p* < 0.05), and prolonged immobility time in FST (*p* < 0.05) (details showed in Figure 3.). Likewise, the defeated rats’ food intake was also reduced, and their body weights were considerably lower when compared pre- and post-social defeat exposure.

### 3.3 YXJYD alleviated depressive-like behaviors in CUMS rats

To validate the antidepressant effect of YXJYD, different concentrations of YXJYD were tested in CUMS-induced depression rats and asssessed the effectiveness by SPT, OFT, and FST. As showed in Figure 3 and Supplementary Table S1, YXJYD improved the CUMS rats’ preference for sucrose in SPT significantly (*p* < 0.05), increased the spontaneous locomotor activity in OFT (*p* < 0.05), and decreased the immobility duration in FST (*p* < 0.05) when compared with the CUMS model group. Along with the decreasing of cortisol in the serum after YXJYD treatment, it is clear that YXJYD had an striking antidepressant effect.

In clinical, it is commonly found adolescent with depression are also suffer from decreasing appetite and losing weight. The weight of model rats in our study was dramatically reduced after 5 weeks of stimulation, which is consistent with similar symptoms in adolescents’ depression. Interestingly, YXJYD treatment help CUMS rats to gain weight significantly (*p* < 0.05), and the rats had a stronger desire to eat after treating with YXJYD. These findings prompted us to investigate the effects of YXJYD on the gut microbiota and metabolites in CUMS-induced depression rats.

### 3.4 Cecal content metabolic profiling and biomarker identification

**FIGURE 4:**
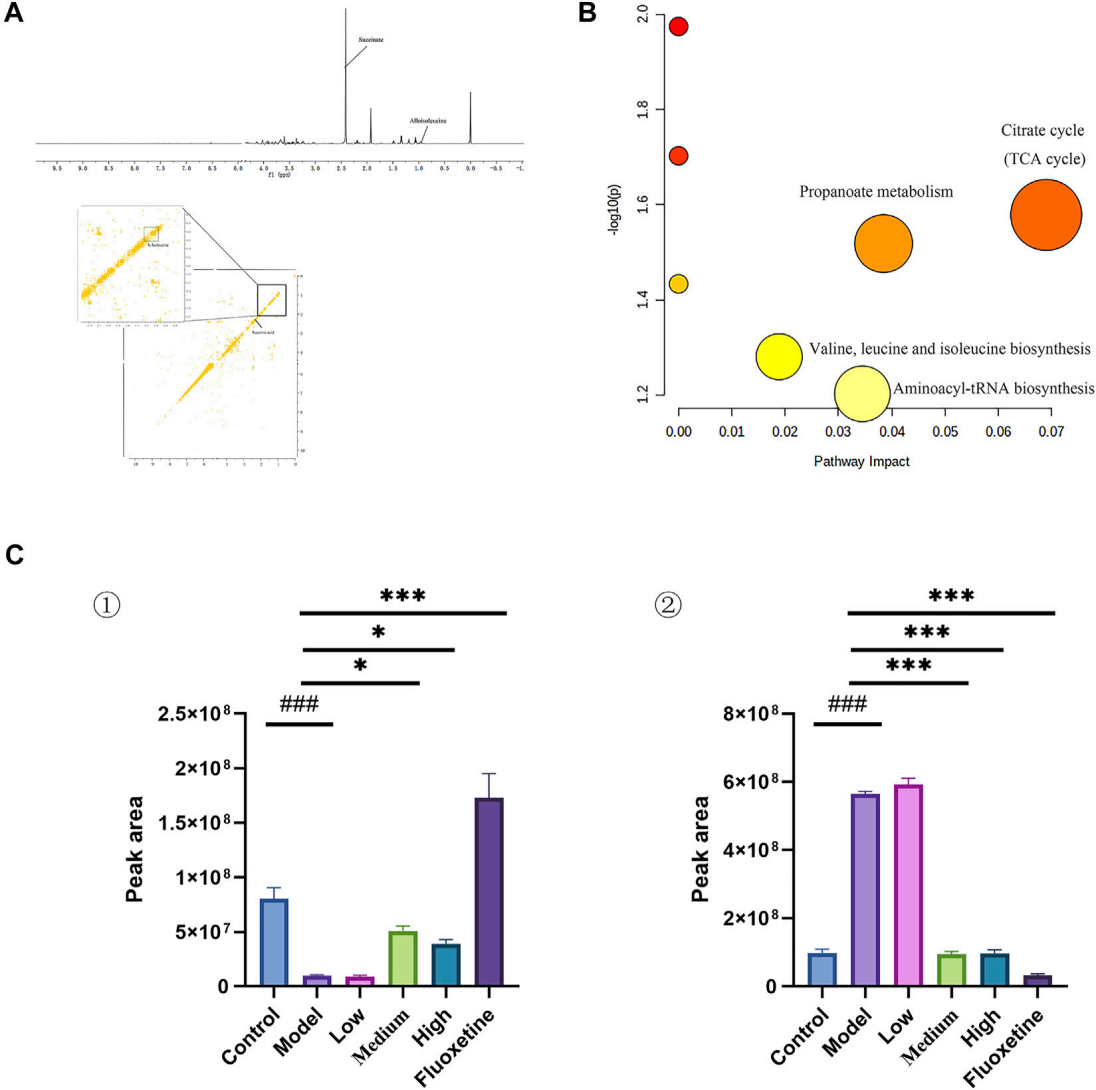
**(A)** 1D and 2D ^1^H-^1^H COSY identification results of different groups of cecal content differential metabolites; **(B)** Enrichment results of metabolic pathways associated with cecal contents; **(C)** Histogram of relative abundance of fecal differential metabolites in depressed rats [L-Isoleucine (①), Succinic acid (②)] (Control: Blank group, Model: Model group, Low: Formulation pellet low dose group, Middle: Formulation pellet medium dose group, High: Formulation pellet high dose administration group. Fluxetine: fluoxetine group) Note: Compared with blank group: ^#^ *p* < 0.05, ^##^ *p* < 0.01, ^###^ *p* < 0.001; Compared with model group: *p < 0.05, **p < 0.01, ***p < 0.001.

Multivariate analysis was performed to assess and compare the changes between control group and CUMS model group to detect biomarker metabolites that associated with the depression. Initially, the unsupervised PCA analysis produced a solution with two significant components explaining 93.4% of the total variance in the data, but the separations between the control group and model group were not obvious. To eliminate the irrelevant noise, we further performed orthogonal partial least-squares discriminant analysis (OPLS-DA) to filter irrelevant information, and obtained a clear separation between the control group and CUMS model group. The internal validation was perfomed to assess the predictive ability of the corresponding OPLS-DA model (*R* ^2^ = 0.996), suggesting that the model was a good fit. To futher evaluate the validity of this model, a random permutation test (200 times) was performed, indicating no overfitting. Then, we calculated the variable importance in the projection (VIP) of the OPLS-DA model to identify the potential biomarkers (Figure 4 and Supplementary Figure S9). The threshold of |VIP|>1, *p* <0.05, and log Fold change > 2 or <0.05 was used to screen candidate metabolites that linked to the development of depression. Finally, the metabolites of L-isoleucine and succinic acid were identified, belonging to energetic metabolism (citrate cycle, propanoate metabolism), amino acid metabolism (Valine, leucine and isoleucine biosynthesis), and genetic information processing (aminoacyl-tRNA biosynthesis) (Figures 4A,B and Table 2). As showed in Figure 4C, compared with the control group, the relative abundance of L-isoleucine in the CUMS model rats was decreased, while that of succinic acid was increased. This is consistent with previous finding reported by Xing et al. (Xing et al., 2015). Interestingly, the abundance of L-isoleucine was increased, and the abundance of succinic acid was decreased after YXJYD treated, suggesting that succinic acid and L-isoleucine may be therapeutic targets of YXJYD.

**TABLE 2:** Qualitative results of potential biomarkers of fecal metabolomics in rats with depression.

No.	Metabolites	Chemical shift (ppm)	VIP	HMDBID	KEGGID
1	L-Isoleucine	0.94(m)	1.13	HMDB0000172	C00407
		1.3(m)	0.69		
2	Succinic acid	2.39(s)	3.46	HMDB0000254	C00042

### 3.5 Urinary metabolic profiling and biomarker identification

**FIGURE 5:**
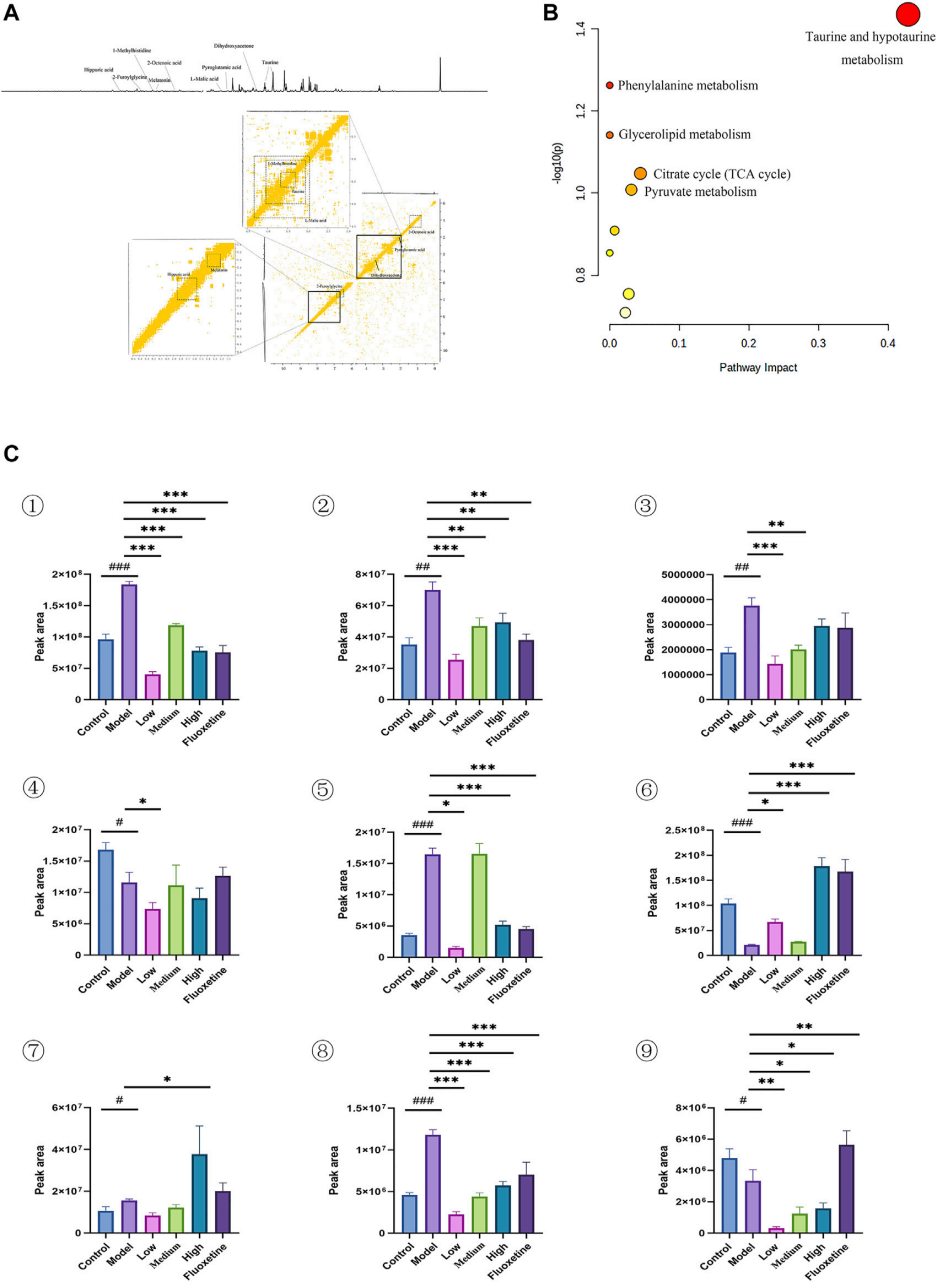
**(A)** 1D and 2D ^1^H-^1^H COSY identification results of different groups of urine differential metabolites; **(B)** Enrichment results of metabolic pathways associated with urine; **(C)** Histogram of relative abundance of urinary differential metabolites in depressed rats [Taurine (1), Dihydroxyacetone (2), L-Malic acid (3), 2-Octenoic acid (4), 1-Methylhistidine (5), Hippuric acid (6) Pyroglutamic acid (7), 2-Melatonin (8), Dimethylglycine (9)] (Control: blank group, Model: model group, Low: formulation pellet low dose group, Middle: formulation pellet medium dose group, High: formulation pellet high dose administration group. Fluxetine: fluoxetine group) Note: Compared with blank group: ^#^ *p* < 0.05, ^##^ *p* < 0.01, ^###^ *p* < 0.001; Compared with model group: *p < 0.05, **p < 0.01, ***p < 0.001.

In order to determine if the difference in the urine metabolome composition can be linked to the action of YXJYD on the gut microbiome, or if they are associated with development of depression, we further to characterize the urinary metabolic profiling and identify biomarker metabolites. Multivariate analytical methods performed on urinary samples were similar to cecal content samples (Supplementary Figure S9). In comparison with control group, nine altered metabolites were identified in serum samples in CUMS model group, including taurine, dihydroxyacetone, L-malic acid, 2-octenoic acid, 1-methylhistidine, hippuric acid, pyroglutamic acid, 2-furoylglycine, melatonin (Figure 5A and Table 3). These metabolites are generated by some microorganisms through energetic metabolism (citrate cycle, pyruvate metabolism, glycerolipid metabolism) and amino acid metabolism (taurine and hypotaurine metabolism, phenylalanine metabolism, glutathione metabolism, tryptophan metabolism) (Figure 5B). Compared with control group, the relative abundance of taurine, dihydroxyacetone, L-malic acid, 1-methylhistidine, pyroglutamic acid, and 2-furoylglycine were upregulated significantly in the CUMS group, while that of 2-octenoic acid, hippuric acid, and melatonin were downregulated significantly in the CUMS group (Figure5C). These findings were consistent with previous researches on comparing depressive persons with healthy person (J. jun Chen et al., 2018). Coincided with our expectation, compared to the CUMS group, the abundance of taurine, dihydroxyacetone, L-malic acid, 1-methylhistidine, and 2-furoylglycine were downregulated significantly after YXJYD treated, while that of hippuric acid and melatonin were upregulated significantly after YXJYD treated. Notably, hippuric acid (also a metabolite of phenylalanine) could be produced by bacterial metabolism in the gut, highlighting the potential involvement of gut microbiota disturbance in the development of depression. These results showed that taurine, dihydroxyacetone, L-malic acid, 1-methylhistidine, 2-furoylglycine, hippuric acid and melatonin were important therapeutic targets of YXJYD.

**TABLE 3:** Qualitative results of potential biomarkers of urinary metabolomics in rats with depression.

No.	Metabolites	Chemical shift(ppm)	VIP	HMDBID	KEGGID
1	Taurine	3.28(t)	0.86	HMDB0000251	C00245
		3.41(t)	1.2		
2	Dihydroxyacetone	3.55(s)	1.28	HMDB0001882	C00184
		2.37(t)	0.59		
3	L-Malic acid	2.67(d)	0.67	HMDB0000156	C00149
		4.28(d)	2.09		
		0.86(t)	0.46		
4	2-Octenoic acid	2.13(q)	0.66	HMDB0000392	NA
		6.64(m)	1.55		
5	1-Methylhistidine	7.03(s)	1.39	HMDB0000001	C01152
		7.80(s)	1.04		
		3.95(d)	0.85		
6	Hippuric acid	7.54(t)	1.18	HMDB0000714	C01586
		7.63(t)	1.1		
		7.82(d)	1.05		
		2.03(m)	0.68		
7	Pyroglutamic acid	2.40(t)	0.64	HMDB0000267	C01879
		2.50(t)	0.61		
		4.14(t)	1.7		
		3.92(s)	0.61		
8	2-Furoylglycine	6.62(s)	1.64	HMDB0000439	NA
		7.17(d)	1.35		
		7.66(s)	1.07		
		1.89(s)	0.71		
		2.93(m)	0.63		
9	Melatonin	3.88(s)	0.98	HMDB0001389	C01598
		6.91(d)	1.48		
		7.21(d)	1.35		
		7.42(d)	1.34		

### 3.6 Serum metabolic profiling and biomarker identification

**FIGURE 6:**
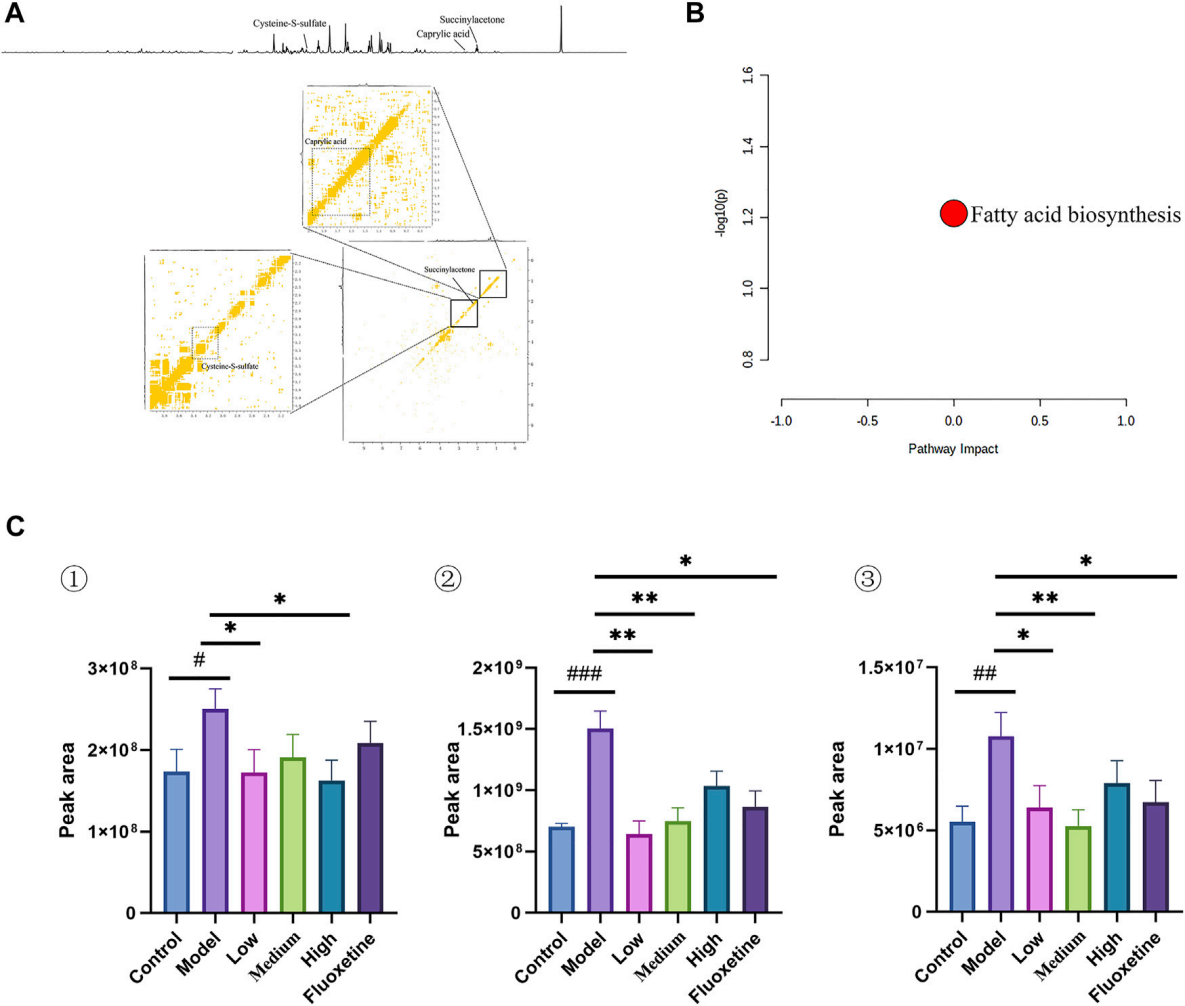
**(A)** 1D and 2D ^1^H-^1^H COSY identification results of different groups of serum differential metabolites; **(B)** Enrichment results of metabolic pathways associated with serum; **(C)** Histogram of relative abundance of serum differential metabolites in depressed rats [Cysteine-S-sulfate (1), Caprylic acid (2), Succinylacetone (3)] (Control: blank group, Model: model group, Low: formulation pellet low dose group, Middle: formulation pellet medium dose group, Fluxetine: fluoxetine group) dose group, High: formulation pellet high dose administration group, Fluxetine: fluoxetine group) Note: Compared with blank group: ^#^ *p* < 0.05, ^##^ *p* < 0.01, ^###^ *p* < 0.001; Compared with model group: * *p* < 0.05, **p < 0.01, ***p < 0.001.

In the serum metabolites, we performed the same multivariate analytical method to identify three significantly altered metabolites between CUMS group and control group, which including caprylic acid, S-sulfo-L-cysteine (cysteine-S-sulfate), and succinylacetone (as showed in Figures 6A,C, and Table 4). These altered metabolites enriched in the pathway of fatty acid biosynthesis (Figure 6B). This result consistent with the finding that fatty acid metabolism is associated with the occurrence and development of depression (Robertson et al., 2017).

**TABLE 4:** Qualitative results of potential biomarkers of serum metabolomics in rats with depression.

NO.	Metabolites	Chemical shift(ppm)	VIP	HMDBID	KEGGID
1	Cysteine-S-sulfate	3.4(s)	1.03	HMDB0000731	C05824
		3.68(d)	2.44		
		4.1(s)	3.15		
		0.89(s)	1.65		
2	Caprylic acid	1.28(t)	4.01	HMDB0000482	C06423
		1.5(s)	0.81		
		2.16(s)	0.87		
		2.26(s)	1.2		
3	Succinylacetone	2.41(s)	1.09	HMDB0000635	NA
		2.81(s)	0.51		

### 3.7 Effect of YXJYD treatment on gut microbiota diversity in CUMS-induced depression rats

**FIGURE 7:**
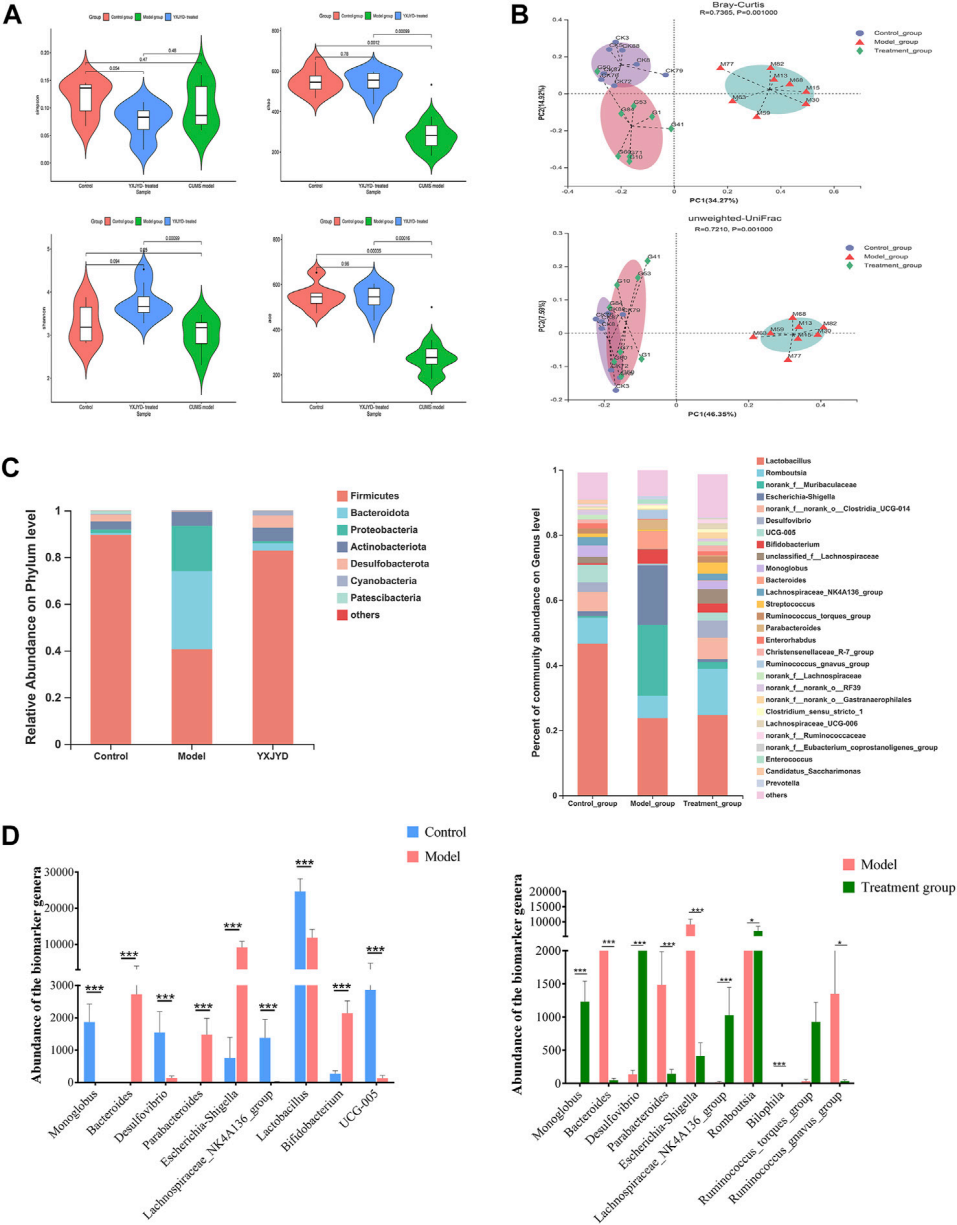
**(A)** Changes in the alpha diversity index (Simpson, Chao, Shannon, Ace) for different groups. Outliers were analyzed based on the Tukey outlier method **(B)** Features of the beta diversity index (Bray–Curtis, unweighted UniFrac) of samples in different groups. **(C)** Gut microbiota abundance of each sample at phylum level and genus level. **(D)** Potential biomarkers of the gut microbiome based on LEfSe analysis among the control, model, and YXJYD groups. (Cecal contents, n; Control = 8, Model = 8, YXJYD = 8).

The distribution of gut microbiota in different groups was compared by sequencing on the bacteria V3-V4 region of the 16S rRNA gene. A total of 1,391,894 reads were generated from twenty-four samples (8 samples per group, respectively). According to the rarefaction curves of all samples, the sequencing data volume is adequate to cover nearly all of microorganisms (Supplementary Figure S4). The Ace and Chao index were calculated to evaluate the α-diversity metrics in different groups (Figure 7A and Supplementary Table S2). As showed in the Figure 7, the index of Ace and Chao were showed markedly difference between control group and model group (*p* < 0.001), whereas showed no significant difference between control group and YXJYD-treated group (*p* = 0.96 and 0.78, respectively). Furtherly, compared with the control group, the Ace index and Chao index were decreased in the CUMS model rats, but that was reversed after YXJYD treated. It is indicated that YXJYD increased microbial richness and diversity, while CUMS caused microbial richness and diversity decreased.

To evaluate the difference of gut microbial composition by pairwise comparison, we performed the beta diversity and principal coordinate analysis (PCoA). The beta diversity analysis revealed that the microbiota of the model group differed considerably between the control group and the YXJYD treatment group (2.1 g/kg). The PCoA plot showed that the model group exhibited significant separation from the other two groups markedly (Figure 7B), indicating a significant difference of gut microbiota composition between model group and control group, whereas YXJYD treatment reduced the difference. The value on the axis records the percentage of results interpreted by each dimension.

### 3.8 Regulation of YXJYD on the microbial distribution at the phylum levels

At the phylum level, the microbiota was dominated by *Firmicutes*, *Bacteroidetes*, *Proteobacteria*, *Actinobacteria*, *Desulfobacterota*, *Cyanobacteria* and *Patescibacteria*, and showed notable differences across control group, model group, and YXJYD high-dose treated group. The community bar plot at the phylum level showed the distributions of the top seven most abundant phyla (Figure 7C). Compared to control group, *Firmicutes* phylum was obviously decreased, while *Bacteroidota* increased in model group, but there is no difference between control group and YXJYD treatment group. Furthermore, Figure 7C demonstrated that CUMS increased the number of *Proteobacteria* and *Actinobacteria* while decreased the abundance of *Desulfobacterota, Cyanobacteria,* and *Patescibacteria*. Notably, a high dosage of YXJYD restored bacterial abundance, and the rats displayed a similar microbial distribution to the control group after YXJYD treatment.

### 3.9 Identified biomarker microbiome at the genus level

**FIGURE 8:**
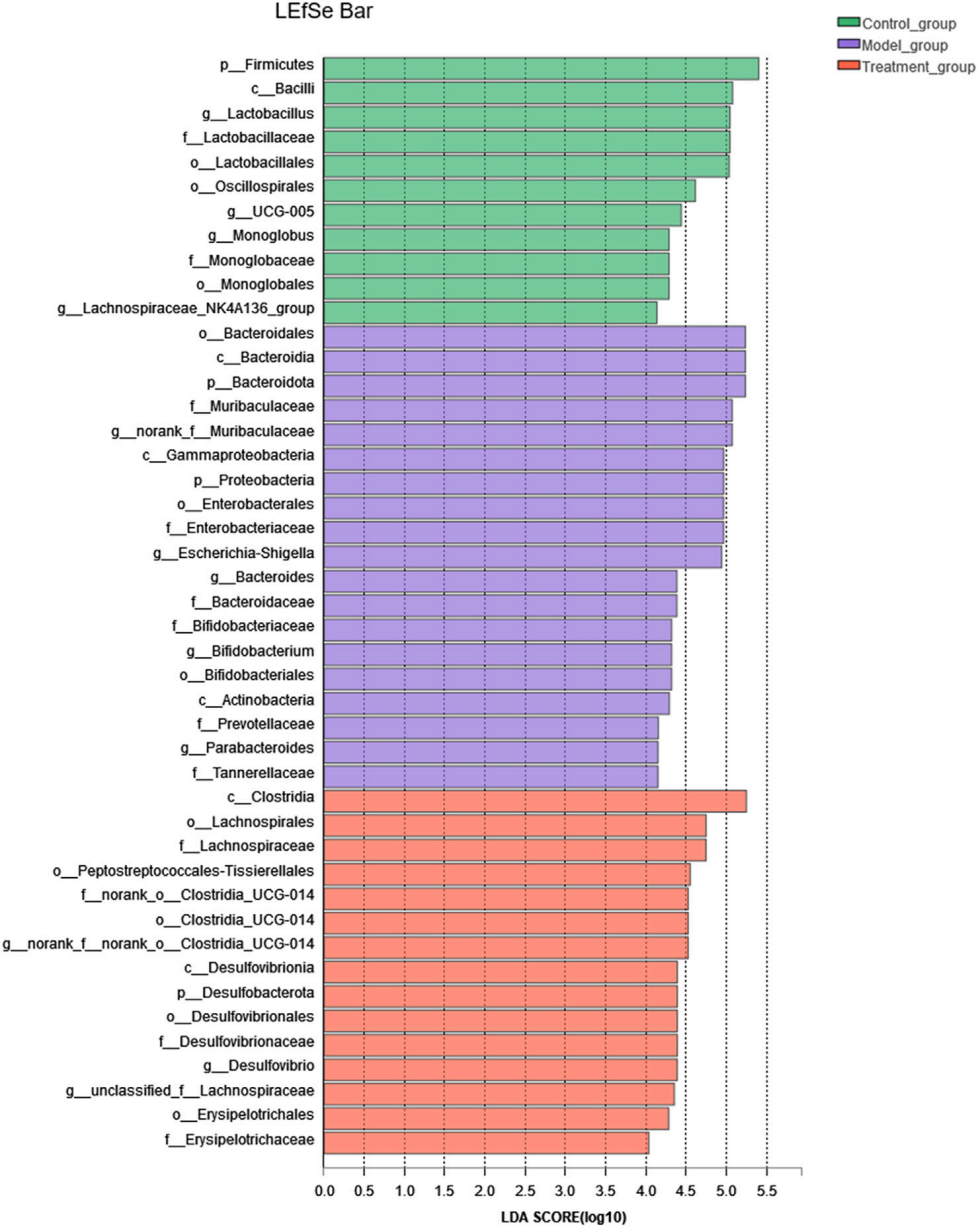
LEfSe analysis of relative abundance of gut microbiome among Control, Model and YXJYD-treatment groups. The larger of the LDA score represents the stronger effect of the species abundance on the differentiation. The Cut-off values were *p* < 0.01 and LDA > 4.

**FIGURE 9:**
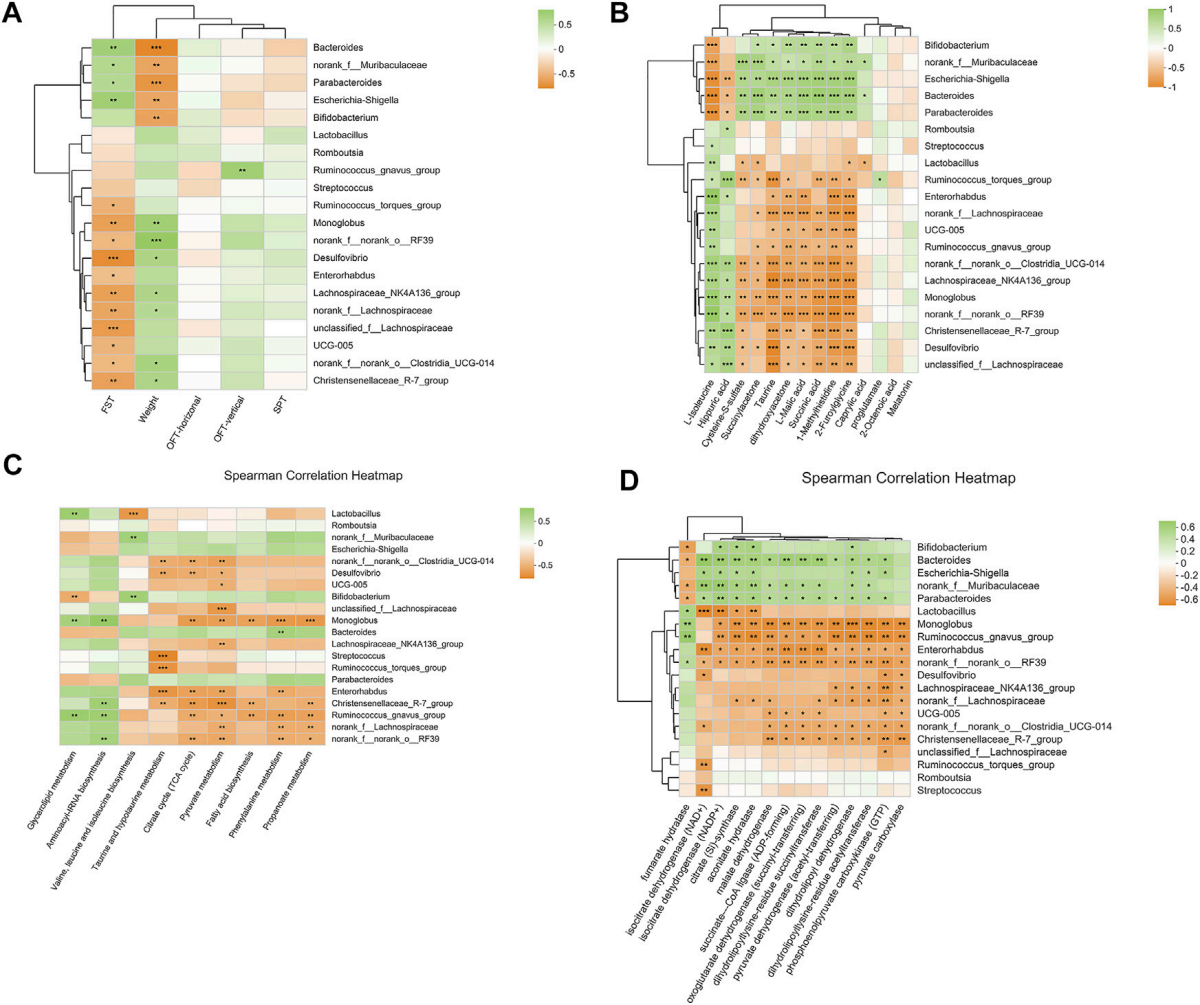
**(A)** Heat map for correlation analysis of intestinal differential microorganisms (relative abundance were rank top of 20) with behaviors; **(B)** Heat map for correlation analysis of intestinal differential microorganisms (relative abundance were rank top of 20) with differential metabolites of urine, cecal content, and serum; **(C)** Heat map for correlation analysis of intestinal differential microorganisms (relative abundance were rank top of 20) with metabolic pathways; **(D)** Heat map for correlation analysis of intestinal differential microorganisms (relative abundance were rank top of 20) with microbial enzymes. *p < 0.05; **p < 0.01; ***p < 0.001. FST, Forced Swimming Test results; OFT, Open Field Test results; SPT, Sucrose Preference Test results.

At the genus level, a variety of microbiotas showed significantly different between model group and control group, including *Lactobacillus*, *Monoglobus*, *Desulfovibrio*, Lachnospiraceae*_NK4A136_group*, *Romboutsia*, *Ruminococc us_torques_group*, and so on (as showed in Figures 7C,D)*.* We further used the method of LEfSe analysis to compare the difference between three groups at the genus level. While performed the LefSe analysis among three groups, with the threshold of logarithmic LDA score 4.0, and *p* < 0.01, the LefSe analysis revealed the genus of *Lactobacillus, Monoglobus, UCG-005,* and Lachnospiraceae*_NK4A136_group* were enriched in the control group, the genus of *Escherichia-Shigella*, *Bifidobacterium, Bacteroides, Parabacteroides* were enriched in the model group (as showed in Figure 8). Besides, as showed in the Supplementary Table S4 from the RF analysis (MDA score > 2.5), 11 key genera for classification were found. Given the results of these two analyses*,* we identified *Monoglobus*, *Bifidobacterium*, *Bacteroides*, *Parabacteroides*, and Lachnospirace ae*_NK4A136_group* were the biomarker genus of depression as well as YXJYD-treatment alteration of microbiota composition. Compared with the control group, *Monoglobus* and Lachnospiraceae*_NK4A136_group* were significantly decreased, while *Bifidobacterium, Bacteroides, Parabacteroides* were significantly increased in the model group. Whereas, compared with the model group, the abundance of *Monoglobus* and Lachnospiraceae*_NK4A136_group* were significantly increased in the YXJYD-treatment group, while that of *Bifidobacterium, Bacteroides, Parabacteroides* were significantly decreased in the YXJYD-treatment group. Furthermore, the abundance of *Monoglobus* was closely correlated with the improvement of behavior symptoms, the variation of metabolites, and hosted metabolic pathways (Figure 9). Based on the above results, we considered that the variation of *Monoglobus* was the most important biomarker genus in the YXJYD-affected alteration of microbiota composition.

### 3.10 Microbial functional predictions and host-microbe interactions

**FIGURE 10:**
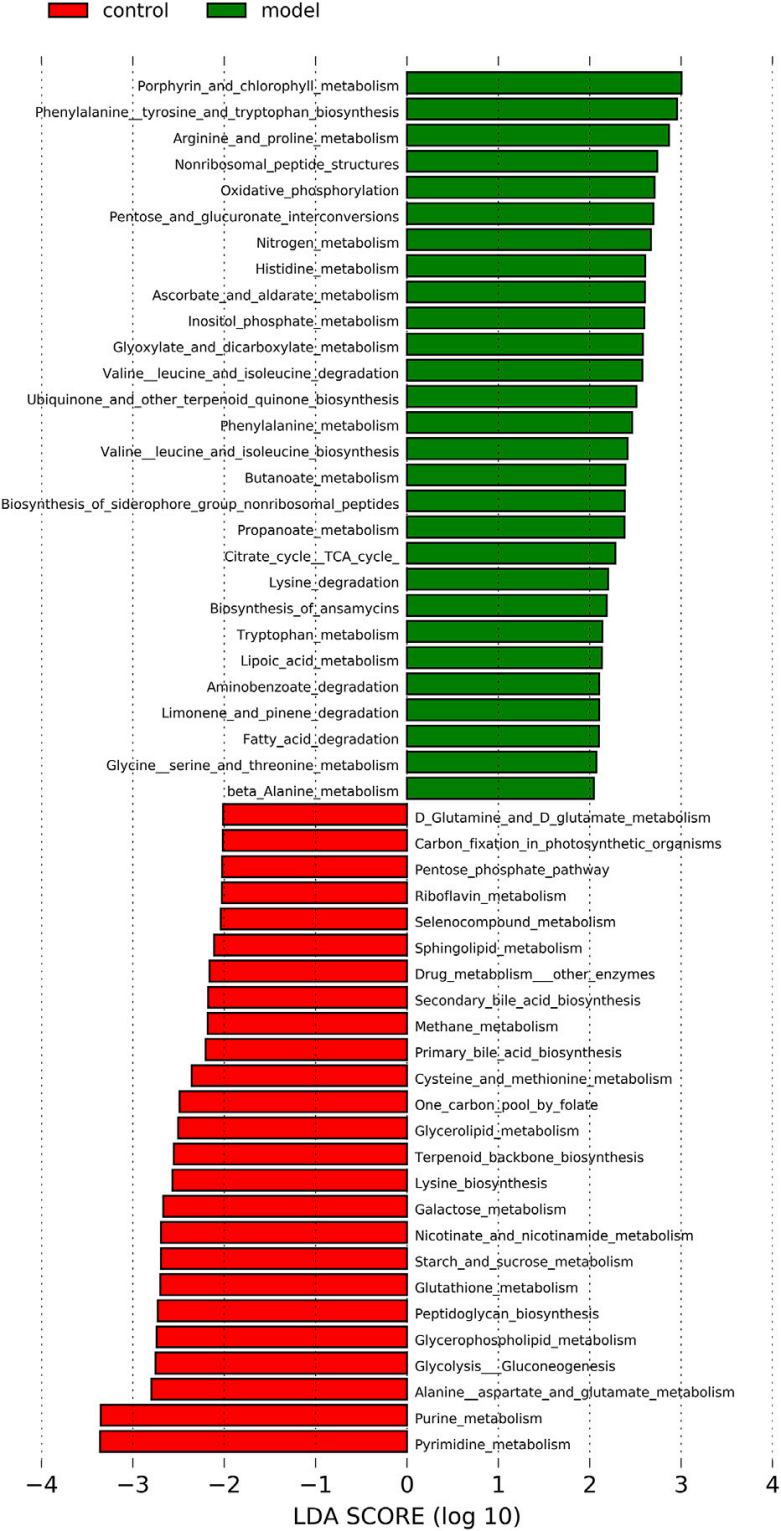
LEfSe analysis of functional metabolism pathways between Control and Model groups. The larger of the LDA score represents the stronger effect of the species abundance on the differentiation. The Cut-off values were *p* < 0.01 and LDA > 2.0.

**FIGURE 11:**
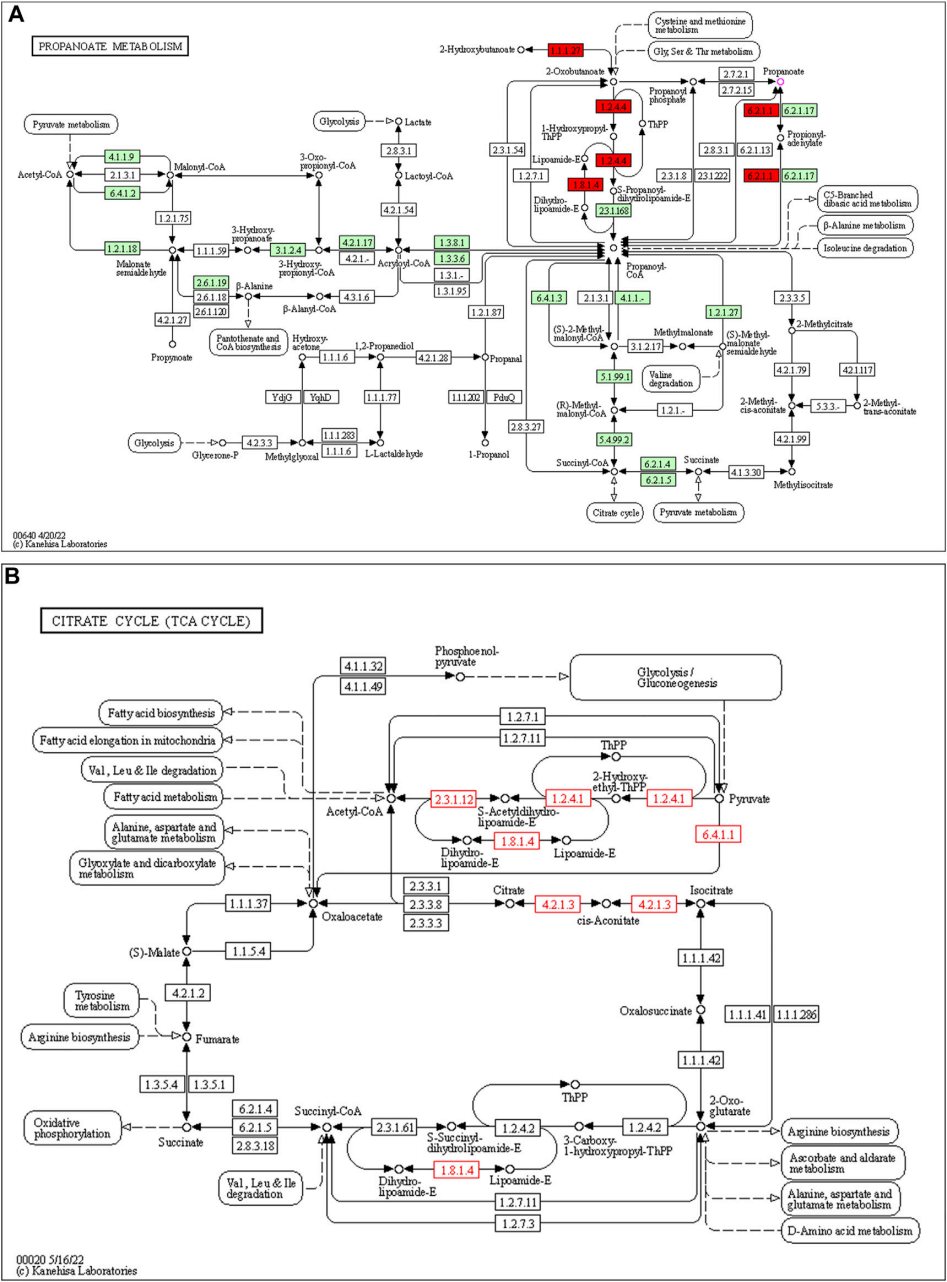
Propanoate metabolism pathway **(A)** and TCA cycle **(B)**. The target enzymes were marked red.

253 differential pathways were predicted by Tax4Fun from levels 3 KEGG orthologs, which included metabolism (53.36%), organismal systems (13.44%), human diseases (16.21%), genetic information processing (7.90%), environmental information processing (4.74%), and cellular processes (4.35%). Among these pathways, we were interested in metabolic pathways, which were linked to depression pathophysiology. To detect the key pathways, LEfSe analysis and RF analysis were performed between the control and model groups. Based on the logarithmic LDA score of 2.0 as the cutoff (Kruskal-Wallis test, *p*-value < 0.01), 54 pathways were identified as significantly differential pathways relevant to depression (Figure 10). In addition, as showed in the plot from the RF analysis (MDA score >2.0) (Supplementary Table S10), 18 key pathways associated with depression were found. Given the results of these two results and the above metabolites related hosted metabolic pathway, citrate cycle and propanoate metabolism were identified as the key metabolic pathways that associated with the occurrence and development of depression. Moreover, 42 enzymes displayed significantly different in the YXJYD-treated groups (Supplementary Table S9). In the propanoate metabolism, there were four enzymes differentially expressed in the YXJYD-treatment group ((*p* < 0.01, LDA > 2)), including alpha-ketoisocaproate dehydrogenase (EC 1.2.4.4), acetyl-CoA synthetase (EC 6.2.1.1), dihydrolipoyl dehydrogenase (EC 1.8.1.4), and L-lactate dehydrogenase (EC 1.1.1.27) (showed in Figure 11A). In the tricarboxylic acid cycle (TCA cycle), there were five differentially expressed enzymes, which included pyruvate dehydrogenase E1 component alpha subunit [EC:1.2.4.1], dihydrolipoamide dehydrogenase [EC:1.8.1.4], pyruvate carboxylase [EC:6.4.1.1], aconitate hydratase [EC:4.2.1.3], and pyruvate dehydrogenase E2 component (dihydrolipoamide acetyltransferase) [EC:2.3.1.12] (showed in Figure 11B).

## 4 Discussion

The present study provided evidence that YXJYD increases the abundance of *Monoglobus* and upregulates four potential metabolic enzymes involvement in propanoate metabolism and five enzymes in TCA cycle. At present, the important role of gut microbiome in the central nervous system development has attracted more and more attention, and accepted by majority that gut microbiome play a critical role in the onset and development of depression via the “microbiota-gut-brain” axis. Thus, target on regulating of gut microbiome may be an effective way for antidepressant. However, available antidepressant drugs may lead, instead, to adverse effect of gastrointestinal disturbances. Hence, how the microbiome is involved in the development of depression has not been systematically investigated.

YXJYD is an empirical prescription for adolescents’depression, and was found to increase appetite and eliminate some digestive disorders from the feedback of adolescents’depressive patients clinically. These feedbacks led us hypothesize that the antidepressant effect of YXJYD may associated with gut microbiota.With an integrated study of the gut microbiota and cecal content, serum, and urine metabolomes, we found that changes in microbiota abundance of the cecal contents significantly contribute to changes in the serum and urine metabolomes, and are linked with reducing depressive-like symptoms in CUMS rats. Our results verified the involvement of gut microbiota metabolism in the effects of YXJYD. Furthermore, the findings demonstrated that the disrupted metabolism was consistent with the pathway enrichment of the changed metabolomes and the projected functional profile of the altered cecal content microbiota in animal models. These findings supported the use of YXJYD as a valuable medication to treat depression in teenagers by modulating their gut microbiotas and host metabolism.

TCM combines well with the healing principle of systemic medicine in the treatment of difficult disorders, such as depression, because of its multi-target and multi-channel characteristics (Maes, Kubera, and Leunis 2008; Maes et al., 2012). Consistent with our expectation, our study indicated that YXJYD improved the behavioral symptoms of depression-like rats in a nondose-dependent manner, and seems superiored to fluoxetine hydrochloride. In addition, 16S rRNA sequencing on the gut microbiota revealed that CUMS decreased the richness and diversity of gut microbiotas significantly, as shown by the Chao and ACE indices. However, it is important to note that the CUMS-induced disruption of gut microorganisms was repaired after treatment with YXJYD. We hypothesized that the antidepressant role of YXJYD may be explained by regulating the “microbiota–gut–brain axis” and then to improve appetite. Evidence is mounting that the gut microbiota plays a critical role in a range of neuropsychiatric illnesses, including depression, schizophrenia, Parkinson’s disease, and autism (Sampson et al., 2016; Sun et al., 2018; Sharon et al., 2019; Zheng et al., 2019). Further, the experimental of transplantation ‘depressed microbiota’ from a depressive patient to healthy animals would induce depressive-like behaviors that offers a foundation for exploring the connections between gut microorganisms and depression. Otherwise, Cai et al. revealed that transferring a healthy person’s fecal microbiota to a depressed patient might alleviate depression-related symptoms by restoring or reconstructing the intestinal microbiota’s composition (Cai et al., 2019). In our study, we found that the richness of *Firmicutes, Cyanobacteria* and *Desulfobacteria* were higher and the richness of *Bacteroidota, Proteobacteria,* and *Actinobacteria* were lower in CUMS model group than that in the control group, which were in agreement with Jiang et al. (H. Jiang et al., 2015) reported that the phylum of *Bacteroidetes, Proteobacteria,* and *Actinobacteria* were positively correlated with depression, while *Firmicutes phylum* was negatively related with depression. Moreover, it is reported that the microbiome members of *Bacteroidete* and *Firmicutes* play crucial roles in modulating host inflammation and immune balance, and a higher F/B ratio is positively associated with the homeostasis of the gut microbiome (Chang et al., 2015). In our study, we observed that *Bacteroidete* and *Firmicutes* were the two major phylas in our samples, and the ratio of *Firmicutes*/*Bacteroidota* in the CUMS model group was significantly lower than that in control group, while there is no significant difference between control group and YXJYD-treated group. It is indicated that CUMS may disturb the balance of the gut microbiota and that finally lead to depression. Otherwise, the ratio of Firmicutes/Bacteroidota was recovered in YXJYD group, indicating that the antidepressant effect of YXJYD was relevant to regulate gut microbiome disturbance.

With further analysis, we identified *Monoglobus* as the key biomarker genera. The abundance *Monoglobus* was decreased in the CUMS model group, but was enriched in the YXJYD-treatment group. We speculate that this genera may be associated with the development of depression. The protective role of *Monoglobus* are supported by previous studies. *Monoglobus* have been shown to be involved in dietary fiber fermentation and associated with healthy communities (C. C. Kim et al., 2019). Moreover, the genus of *Bacteroides, Parabacteroides, Escherichia-Shigella, Bifidobacterium,* and *Ruminococcus_gnavus_group* were significantly downregulated in the YXJYD group, while *Lactobacillus, Desulfovibrio,* Lachnospiraceae*_NK4A136_group, Romboutsia, Bilophila,* and *Ruminococcus_torques_group* were not. Consistent with this finding, higher abundance of *Bacteroides* and lower abundance of *Lactobacillus* were also found in the feces from patients with major depressive disorders (MDD), and previous investigations widely reported that upregulation of *Bacteroides* linked with higher peripheral cytokine levels and increased inflammation in MDD (Yang et al., 2020). The highest LDA score of *Lactobacillus* indicated that the reduced *Lactobacillus* was the most prominent biomarker genus in the CUMS-induced alteration of microbiota composition. Reduced level of gut *Lactobacillus* has also been observed in people with MDD, and that partly because stressor exposure associated with the colonic mucosa, reducing *Lactobacillus* abundance (Heym et al., 2019). Moreover, the anti-inflammatory activity of the *Lactobacillus* genus has been shown to improve cognitive function (Aizawa et al., 2016; J. K. Kim et al., 2020). Alternatively, our study also observed a higher *Bifidobacterium* in CUMS-induced depressive rats. Previous studies have been reported mono-colonisation of mice with human *Bifidobacterium-*rich microbiota had higher proinflammatory Th17 intestinal cells compared to mice colonised with *Bifidobacterium*-depleted microbiota (Ang et al., 2020) (Wang et al., 2014). Furthermore, a higher abundance of *Romboutsia* and *Bilophila* were also displayed in our study. *Romboutsia,* known SCFA producers, has been confirmed to be strongly correlated with the butyrate levels, while fecal butyrate was negatively correlated with depressive symptoms in PD patients.

It may be explained that YXJYD could affect several pathogenic targets concurrently with multiple botanical drugs. We performed HPLC-MS/MS to analyze the chemical compounds of YXJYD and identified 23 compounds enriched in the YXJYD. The majority of the compounds were found to have considerable antidepressant properties. Ginsenoside Rb1, for example, has been shown to have potential antidepressant-like effects in depression model mice by increasing BDNF signaling and encouraging hippocampus neurogenesis. (N. Jiang et al., 2021); Ginsenoside Rg3 was also reported to be effective in ameliorating depressive-like behavior by regulating microglia activation and nuclear factor kappa B (NF-κB) pathway (Kang et al., 2017); Ginsenoside Rf significantly restored depression-like behavioral abnormalities by increasing glial fibrillary acidic protein (GFAP) expression and decreasing Ki-67 expression in the PFC, as well as reducing astroglial changes in the hippocampus (Y. Kim et al., 2020); Magnoflorine has been shown to have an antidepressant effect in brain tissue by increasing the expression of lysine specific demethylase 1 (LSD1), despite its poor liposolubility and low permeability (Barua et al., 2018). Because YXJYD contains numerous components, each of which exerts a separate mechanism, determining the antidepressant mechanism of YXJYD is difficult.

Metabolite is the end products of biological processes, and it may display physiological circumstances directly due to its “downstream” regulatory relationships with the genome, transcriptome, and proteome levels (Bi et al., 2021). Furthermore, metabolites serve as important messengers between the gut microbiota and the host. Previous research has shown that the gut microbiota influences the onset and development of depressive-like behaviors via a metabolic route regulated by the host (P. Zheng et al., 2016). Therefore, we sought to uncover the metabolic mechanism behind the flow of metabolites from gut to brain for reliving depressive mood and behaviors by analyzing the changes of metabolites in serum, urine, and cecal contents. According to the previous metabonomic researches on depression, remarkable changes of metabolites were mostly enriched in energy metabolism, amino acid metabolism, gut microflora metabolism and amino acid neurotransmitters (S. Zheng et al., 2010; Xing et al., 2015; Gao et al., 2014). Consistent with these reports, a number of amino acid neurotransmitters and their derivatives, including taurine, pyroglutamic acid, 2-furoylglycine, and hippuric acid, were considerably changed in urine between CUMS model rats and control rats. Moreover, we found that these altered amino acid neurotransmitters enriched in the amino acid metabolism pathway of taurine and hypotaurine metabolism, phenylalanine metabolism, and glutathione metabolism. These results are in line with the findings of a systematic analyse that indicated abnormalities of amino acid neurotransmitters in depression models (Pu et al., 2021).

Meanwhile, a significant lower level of melatonin was found in CUMS-induced depression rats and was upregulated after treatment with YXJYD. Melatonin is a hormone generated by the pineal gland from serotonin (5-HT) as a precursor via tryptophan metabolism. Other authors using different classes of antidepressants have also found that antidepressants increased melatonin levels, included imipramine (Miller et al., 2001), clomipramine (Rabe-Jabłońska and Anna, 2001), desipramine (Kennedy and Brown 1992), clorgyline, tranylcypromine and phenelzine (Carvalho et al., 2009). Therefore, increased of melatonin levels seems to be an indicator of effective antidepressant. The level of melatonin was elevated in rats after treating with YXJYD. It is suggesting that the antidepressant effect of YXJYD may be associated with regulation of tryptophan metabolism. In comparison with control group, lower levels of succinic acid and L-malic acid were found in CUMS model rats, which are significant biological molecules and crucial intermediates in the TCA cycle linked to glycometabolism and energy metabolism. This is also consistent with our final results that we identified the pathway of TCA cycle and propanoate metabolism as the key pathways relevant to YXJYD treatment. Furthermore, succinic acid is a short-chain fatty acid that is essential for energy transmission and the Krebs cycle. Succinic acid levels was rose following CUMS, which was consistent with earlier research (Xing et al., 2015)*.* As a result, these changed metabolites suggested that antidepressant effect of YXJYD may be linked to correct energy metabolism imbalances, as previously described, and that providing extra creatine or ATP may have antidepressant action (Cao et al., 2013; Cunha et al., 2018). Besides, succinic acid is crucial for energy transportation and is also an important factor in fatigue, as well as hippuric acid (HA) is the metabolite of phenylalanine (Phe) metabolized by the gut microflora, suggested that there was a significant effect on the gut by YXJYD treatment and then improved the fatigue as feedback from adolescents’depressive patients in clinical (Ladep et al., 2006).

Furthermore, the current findings revealed that L-isoleucine levels were reduced in the depression model and that YXJYD considerably increased L-isoleucine levels. It is reported that L-isoleucine affected the mammalian central nervous system (CNS) by two ways. Firstly, isoleucine can be used as a significant amino group donor for the creation of brain glutamate, a crucial CNS neurotransmitter, after it has quickly crossed the blood–brain barrier (Ni et al., 2008). Impaired glutamate homoeostasis and glutamatergic neurotransmission have been identified as the primary causes of depression’s incidence and progression (Shao et al., 2013; G. Chen et al., 2015). Second, the dipeptide glutamine–isoleucine has been found to greatly enhance locomotor activity in rats by injecting this neuropeptide into the ventral tegmental region of the rat midbrain, which transmits afferent GABAergic and glutamatergic projections to the PFC (Berberian, Sanchez, and Celis 2002). Isoleucine acts as essential ketogenic and glucogenic amino acid to play a significant role in energy metabolism. Isoleucine may be transformed into succinyl-CoA via transamination with alpha-ketoglutarate during the tricarboxylic acid cycle (TCA) oxidation process, or it can be turned into oxaloacetate for gluconeogenesis. These findings revealed that modulating L-isoleucine levels and TCA oxidation or gluconeogenesis might be a promising treatment target for depression, and the antidepressant mechanism of YXJYD was connected with regulating neurotransmitter biosynthesis and energy metabolism (G. Chen et al., 2015).

## 5 Conclusion

Our current results demonstrated that antidepressant effect of YXJYD was evident in CUMS rats and it’s mechanism possibly be associated with increasing the abundance of *Monoglobus* and upregulating enzymes involvement in propanoate metabolism and in TCA cycle. In comparison to other antidepressant drugs, the concentration of YXJYD is lesser but showed a striking effect. Coupling with the efficacy for adolescents in clinical, YXJYD would be a promising antidepressants for further study. This work will make a great contribution to the development and application of YXJYD for the treatment on adolescents’ depression. Moreover, there are still some limitations in this study. First of all, the microbiology we used can only comprehend the relative abundance of intestinal microbiota, not the exact composition. Secondly, the potential molecular mechanism of the effect of YXJYD on gut microbiota in this study needs to be further validated. Finally, this study only involved the regulation of gut microbiota and body’s metabolites by YXJYD, and the linked between gut and brain that can be described was limited. It is valuable for us to explore the molecular mechanism of YXJYD by the gut-brain axis in further study.

## Data Availability

The datasets presented in this study can be found in online repositories. The names of the repository/repositories and accession number(s) can be found below: NCBI BioProject, PRJNA862250.
