# Supplementary material for: Integrated 16S rRNA sequencing and metabolomics analysis to investigate the antidepressant role of Yang-Xin-Jie-Yu decoction on microbe-gut-metabolite in chronic unpredictable mild stress-induced depression rat model
Source: Front Pharmacol. 2022 Sep 30;13:972351. doi: 10.3389/fphar.2022.972351 (PMC9565485; doi:10.3389/fphar.2022.972351)
Supplement: Supplementary file 2 [file DataSheet1.docx]

Supplementary Material

# Supplementary Figures and Tables

## Supplementary Flies

**Table S1.** Original data table of behavior test experiments. Cut-off values of two-way ANOVA p < 0.05.

**Table S2.** Statistical analysis of alpha diversity index of different groups. Cut-off values of Kruskal-Wallis test with FDR method of Benjamini and Hochberg was FDR < 0.05.

**Table S3.** LEfSe analysis of taxonomic composition between Model group and Treatment group. Cut-off value of Kruskal-Wallis test was p < 0.01 with a threshold of LDA > 4.

**Table S4.** Random forest (RF) analysis result of the microbial between model group and control group.

**Table S5.** Spearman correlation analysis between microbiota (genus level) and behavior test results.

**Table S6.** Spearman correlation analysis between microbiota (genus level) and metabolites.

**Table S7.** Microbial KEGG pathways predicted by the Tax4Fun.

**Table S8.** LEfSe analysis of microbial metabolic pathways based on KEGG pathway among every three groups. Cut-off value of Kruskal-Wallis test was p < 0.05 with a threshold of LDA > 2.

**Table S9.** LEfSe analysis of microbial metabolic enzyme based on KEGG pathway among every three groups. Cut-off value of Kruskal-Wallis test was p < 0.05 with a threshold of LDA > 2.

**Table S10.** Random forest (RF) analysis result of the functional pathways between model group and control group.

## Supplementary Figures

**1.DL-Arginine**


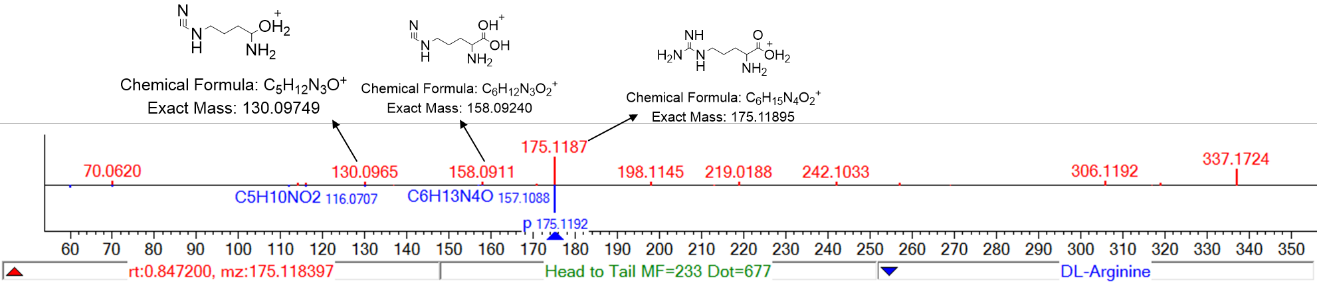


**2.Synephrine**


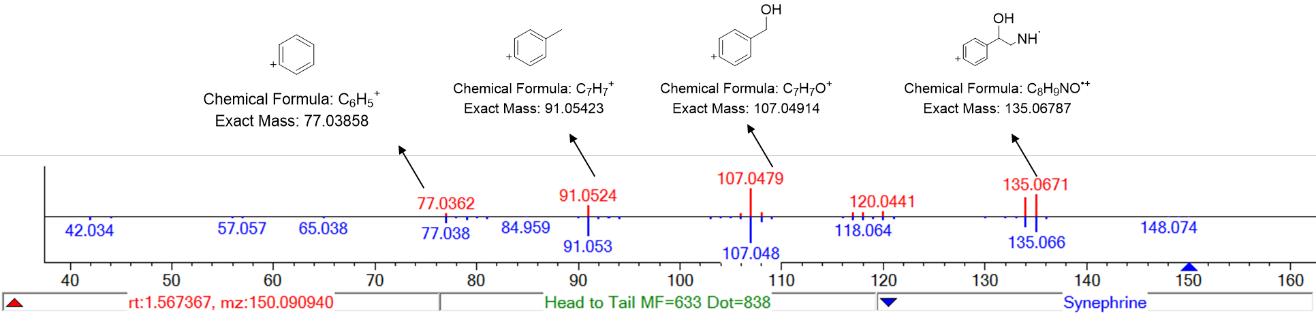


**3. Magnoflorine**


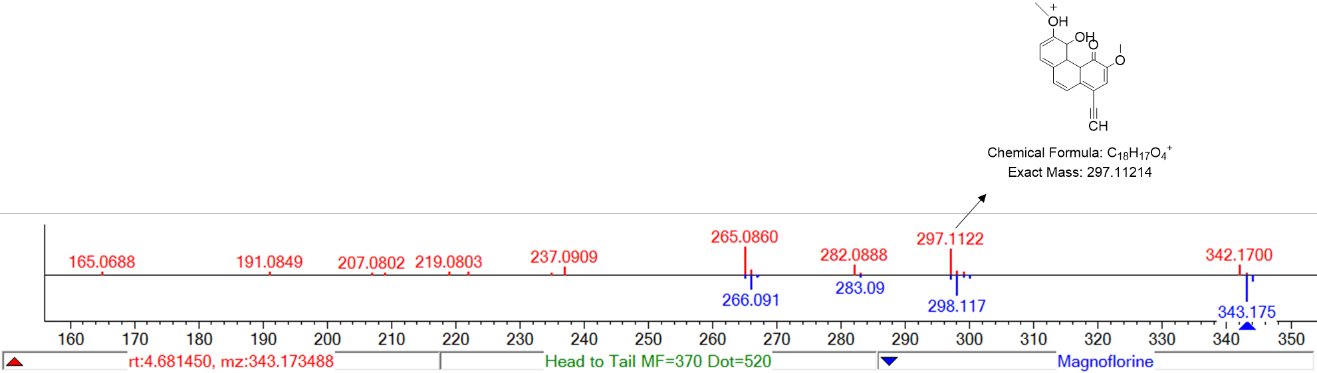


**4. Naringenin**


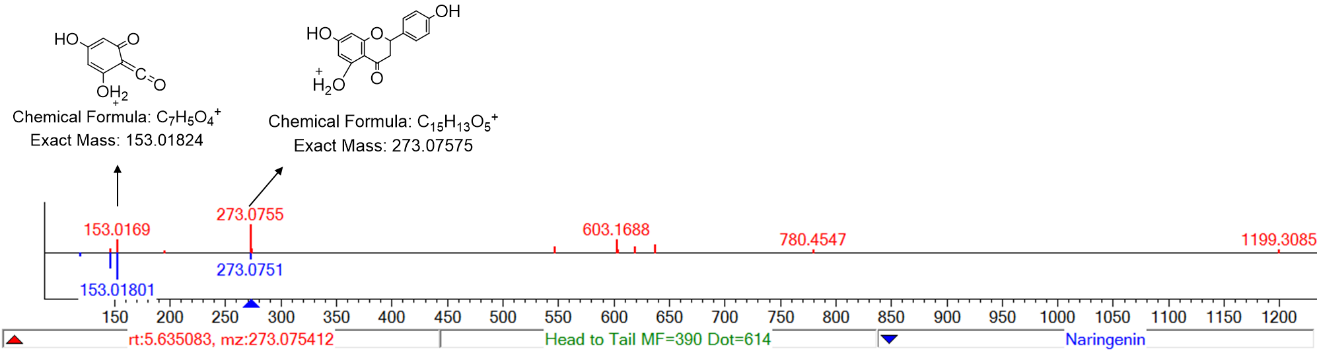


**5. Quercetin**


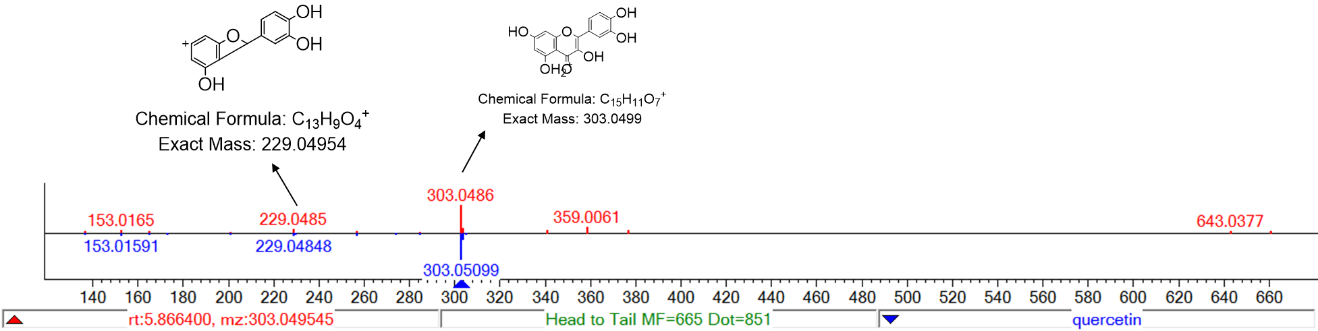


**6. Baohuoside V**


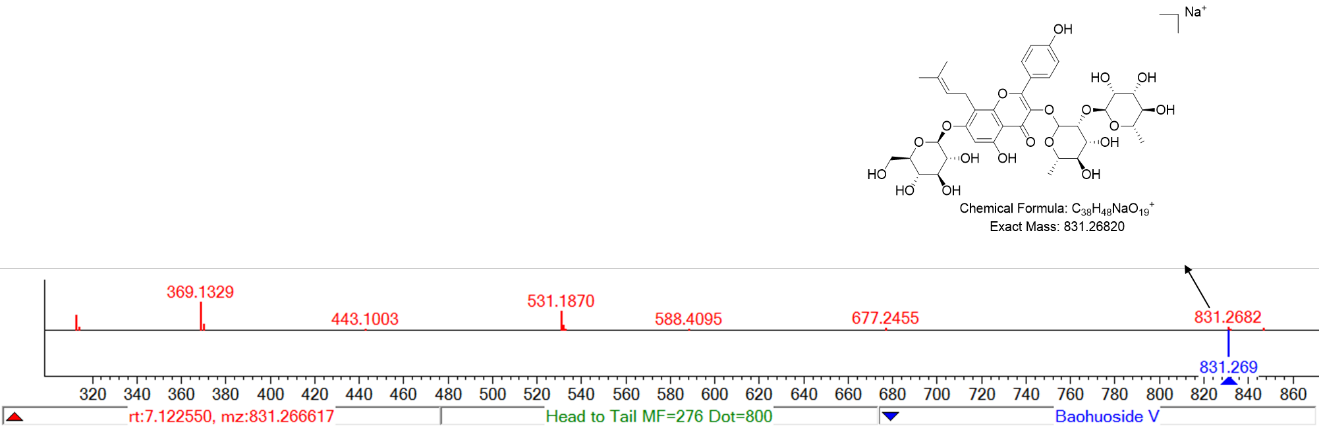


**7. Epimedin B**


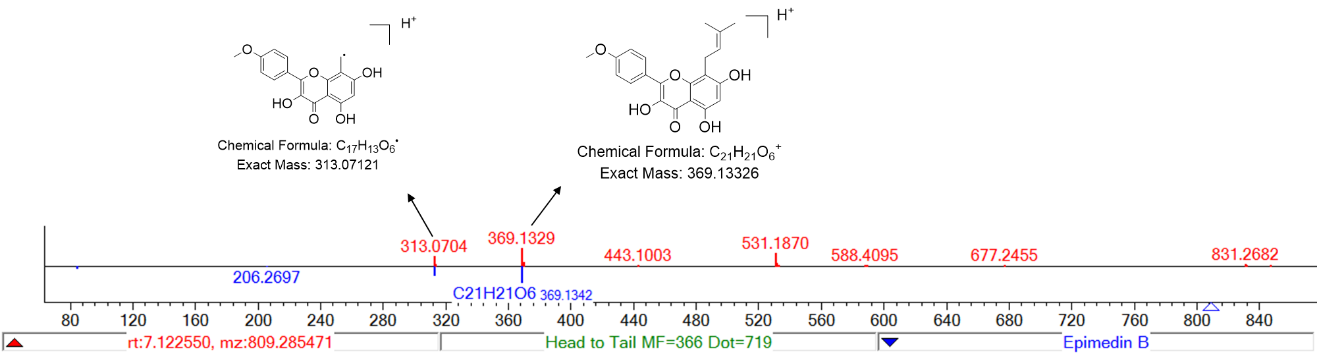


**8. Epimedin C**


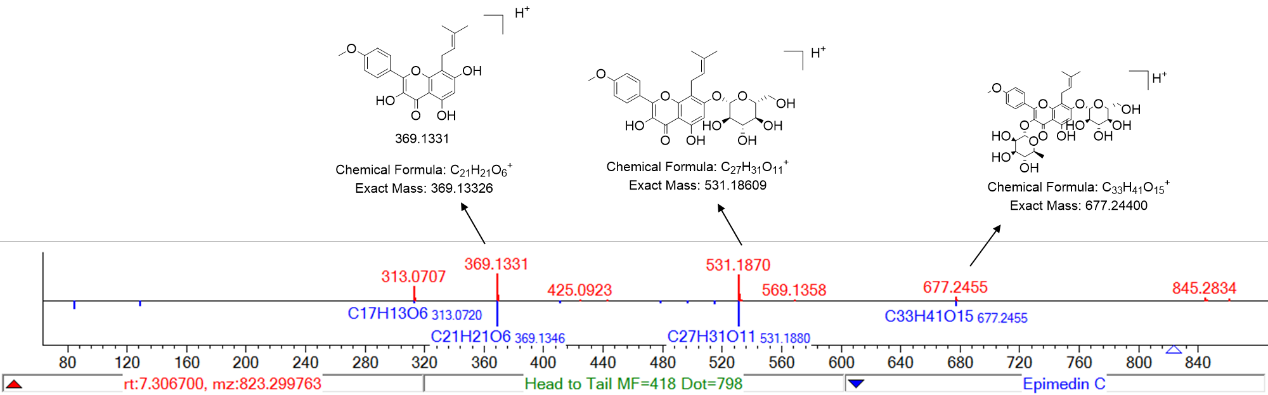


**9. Icariin**


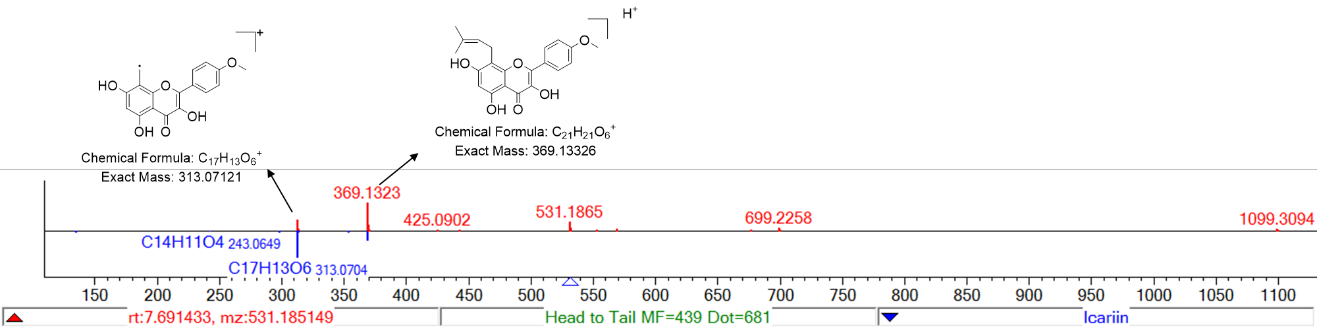


**10. Icariside II**

**
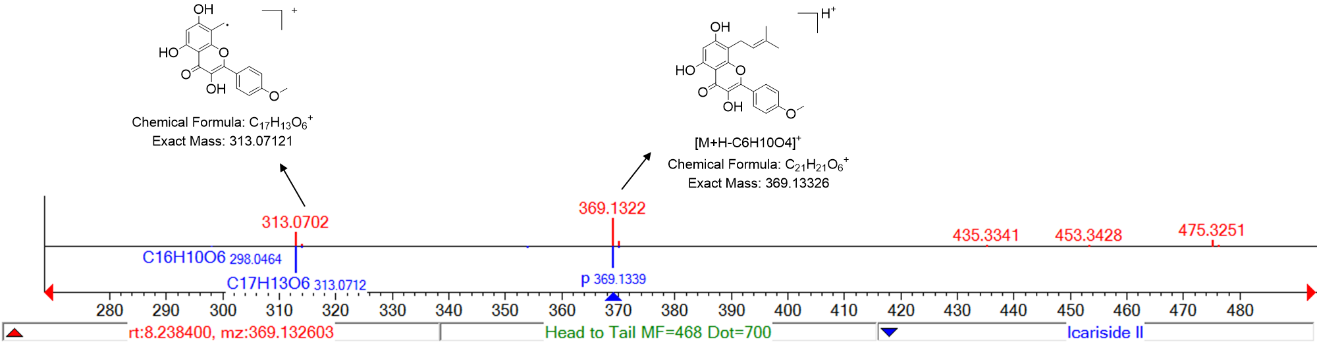
**

**11. Gardenin**


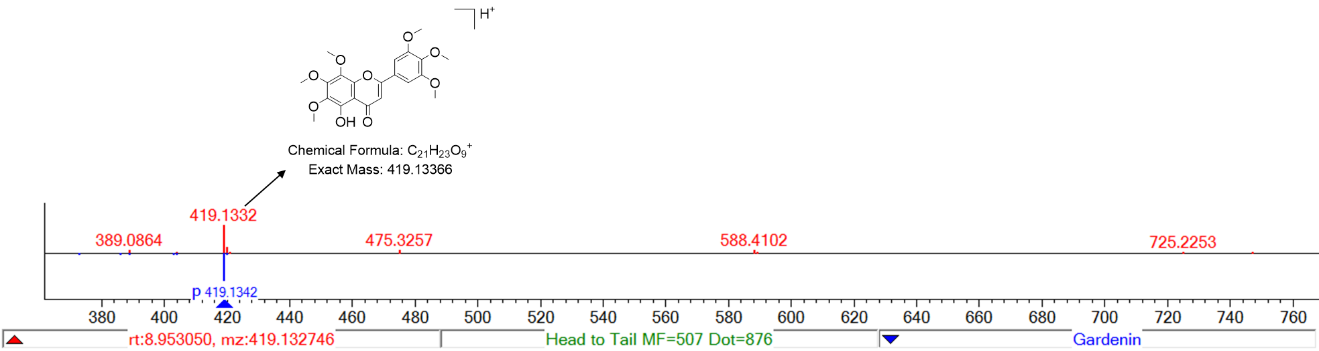


**12. Isoanhydroicaritin**


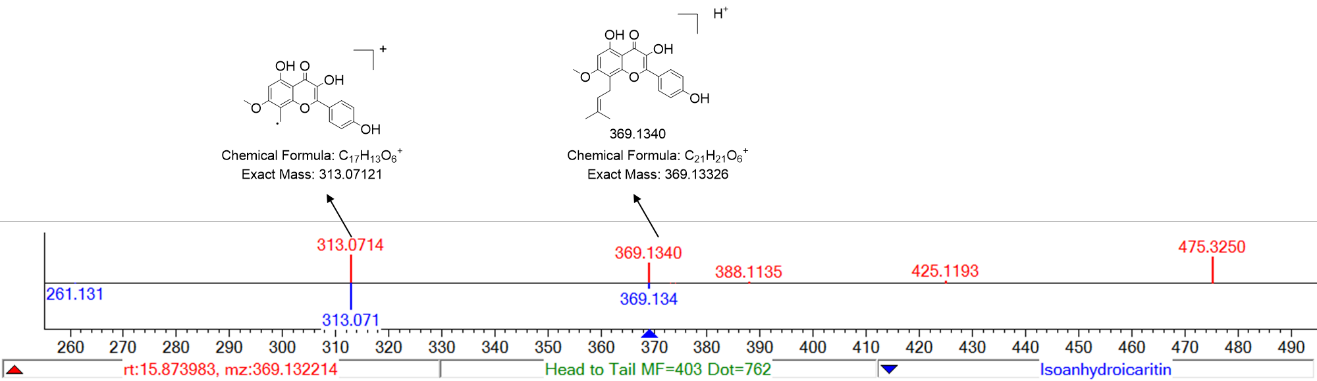


**13. Schizandrol A**


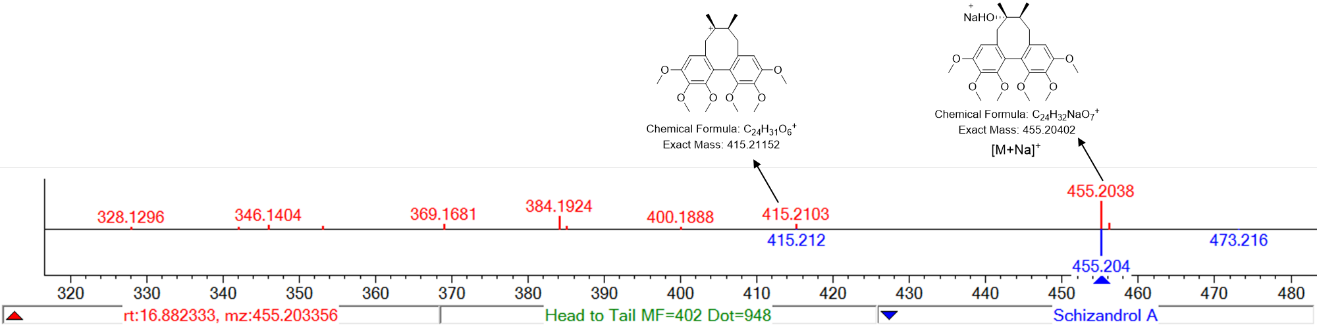


**14. Schizandrin**


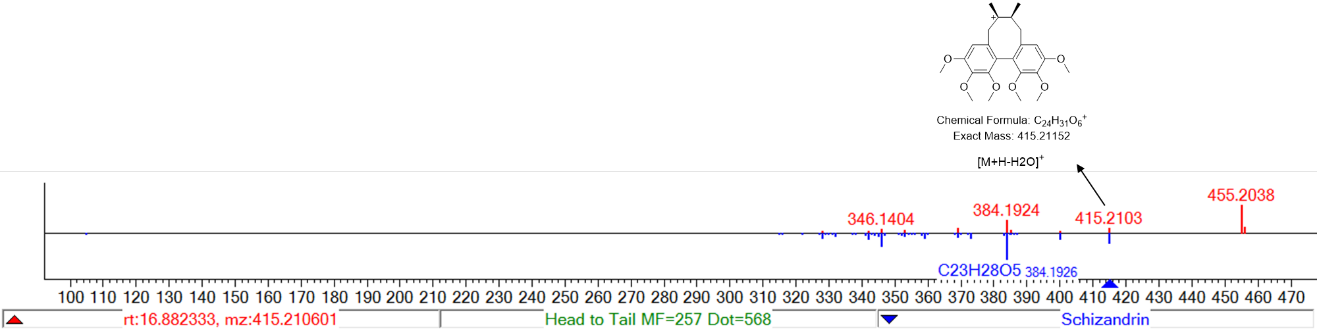


**15. Nobiletin**

**
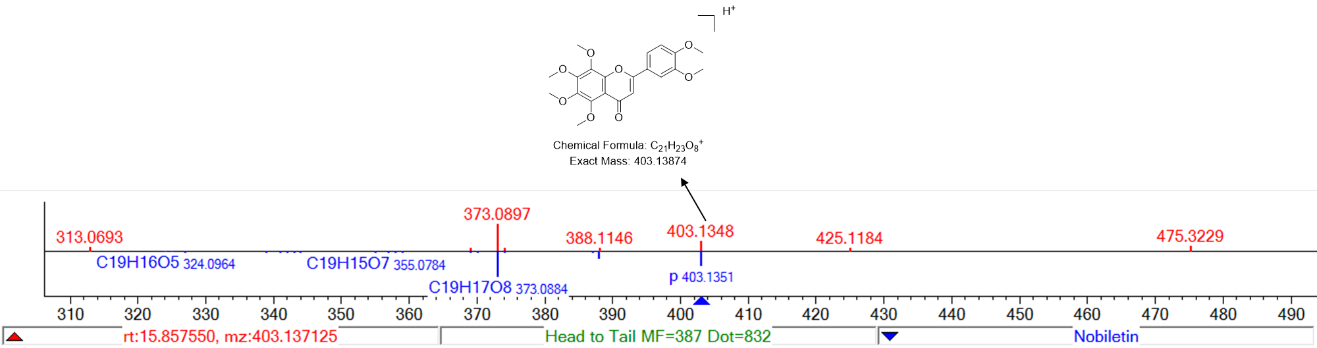
**

**Figure S1.** EIC chromatogram and MS^2^ spectra of each compound in ESI (+).

**1.Eriodictyol-7-O-rutinoside**


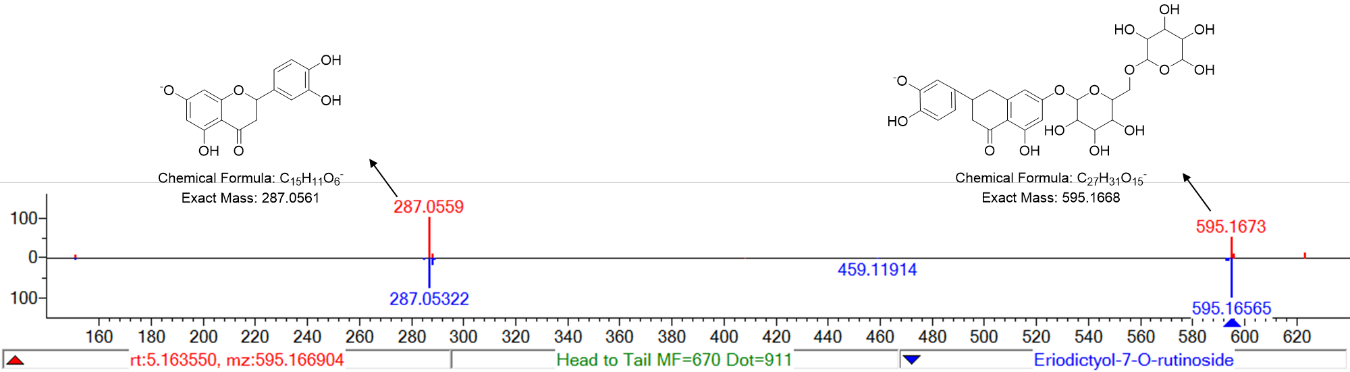


**2. Hyperoside**


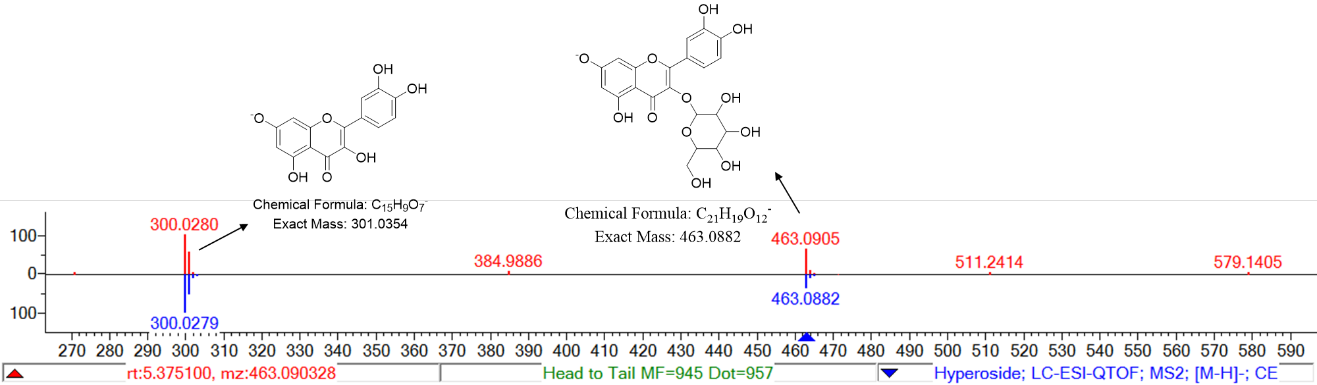


**3. Isoquercetrin**


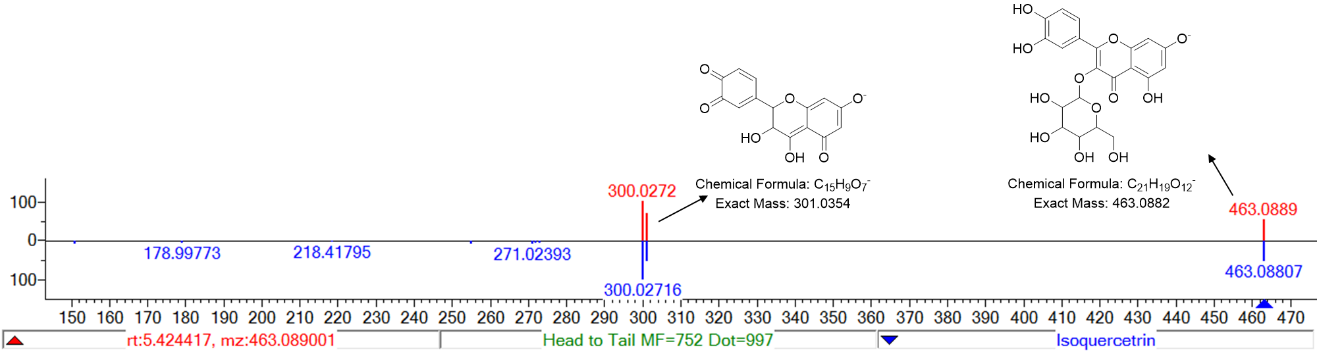


**4. Naringenin-7-O-rutinoside**


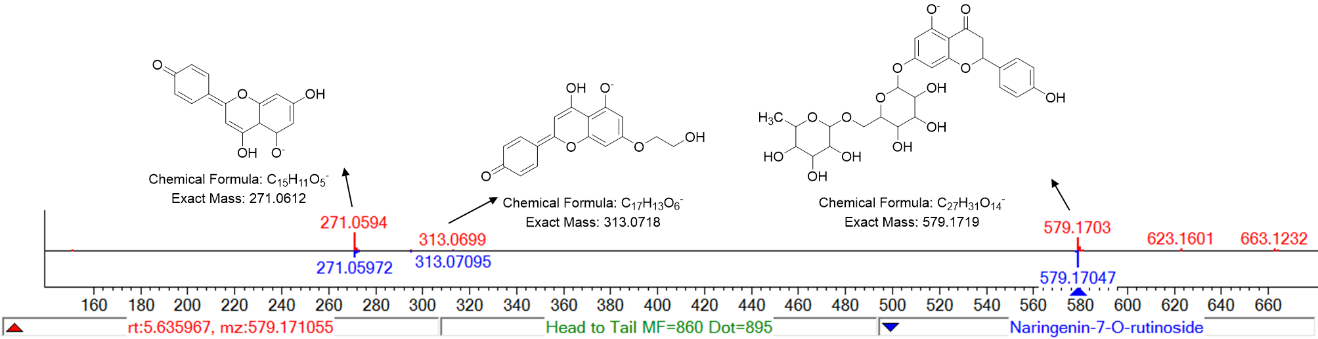


**5. Epimedin B**


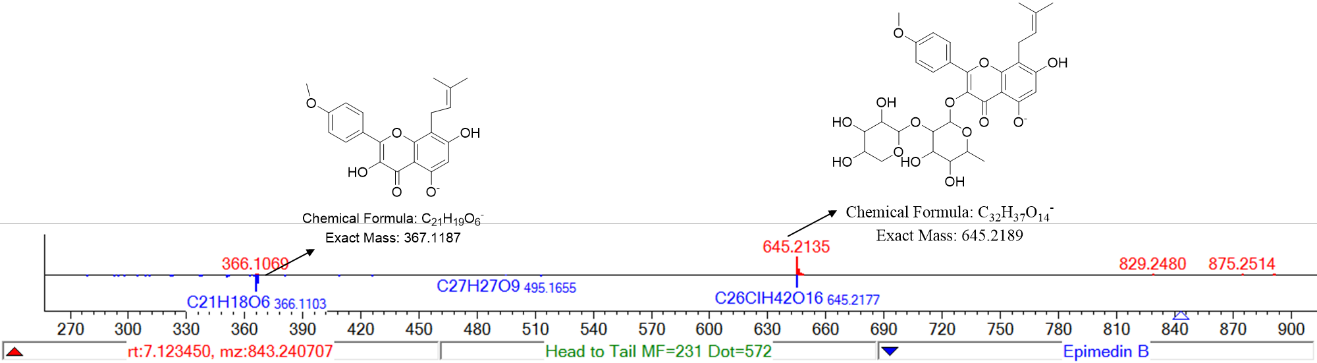


**6. Epimedin C**


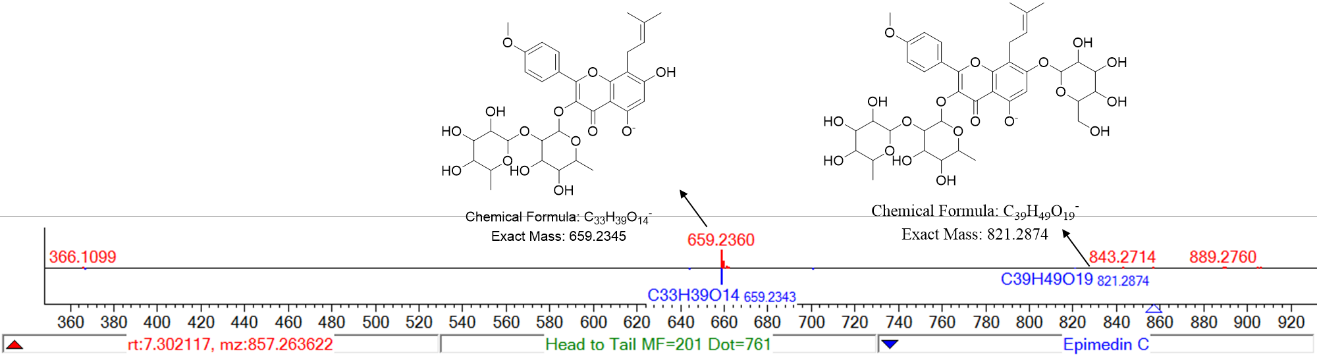


**7. Icariin**


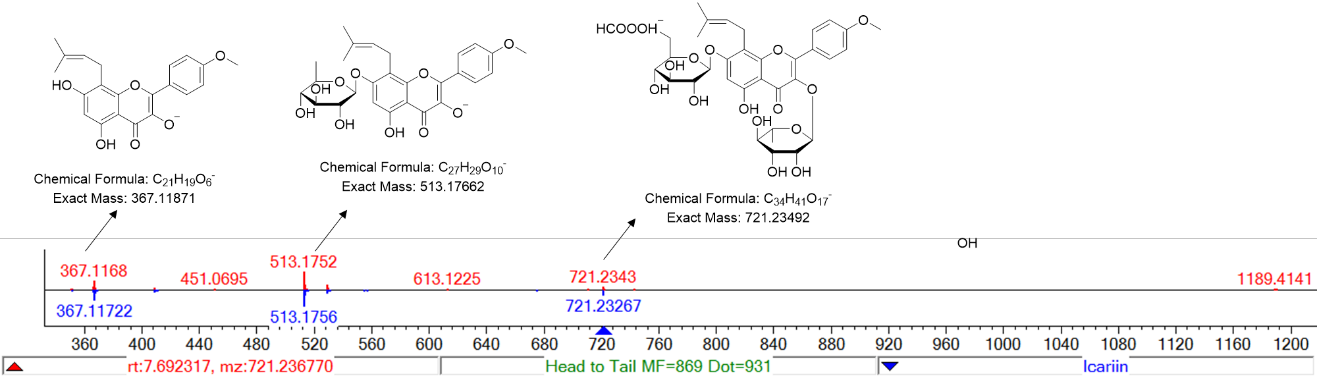


**8. Icariside II**


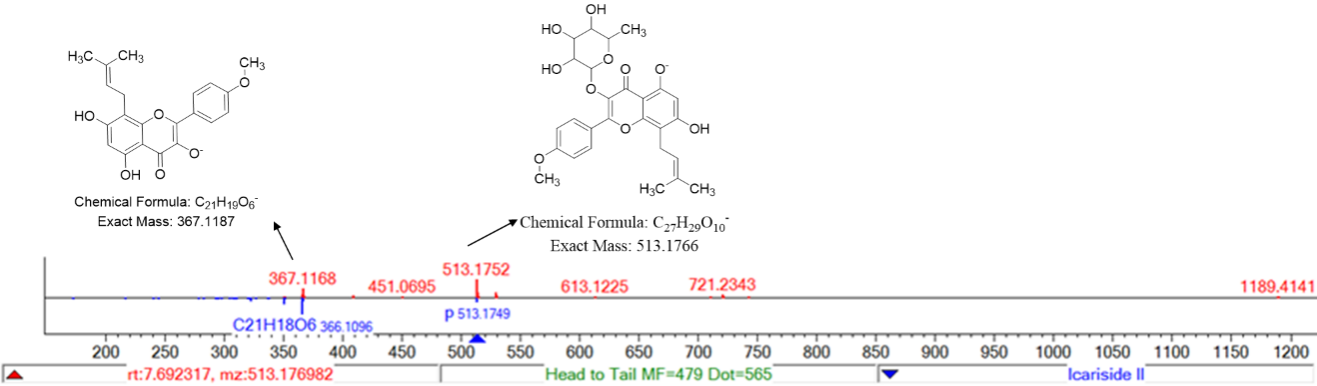


**9. Icariside I**


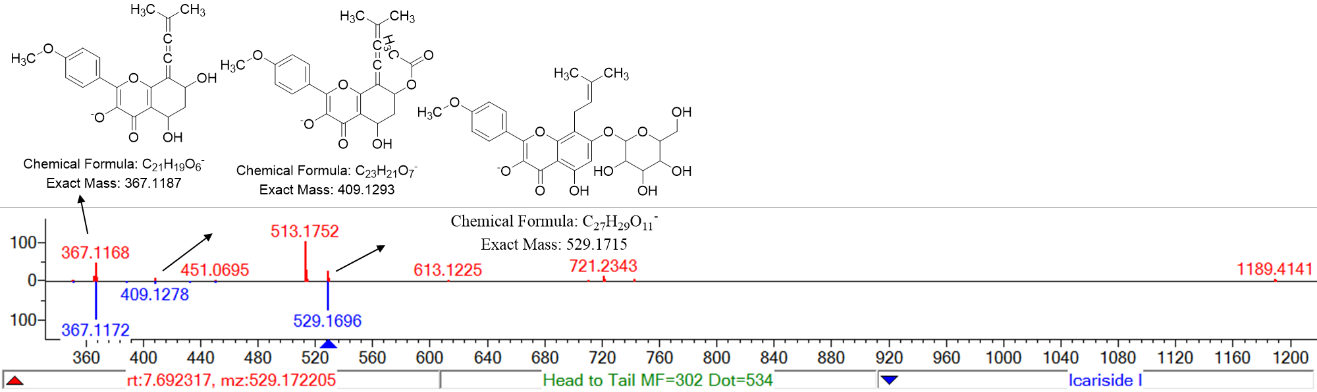


**10. Ginsenoside Rf**


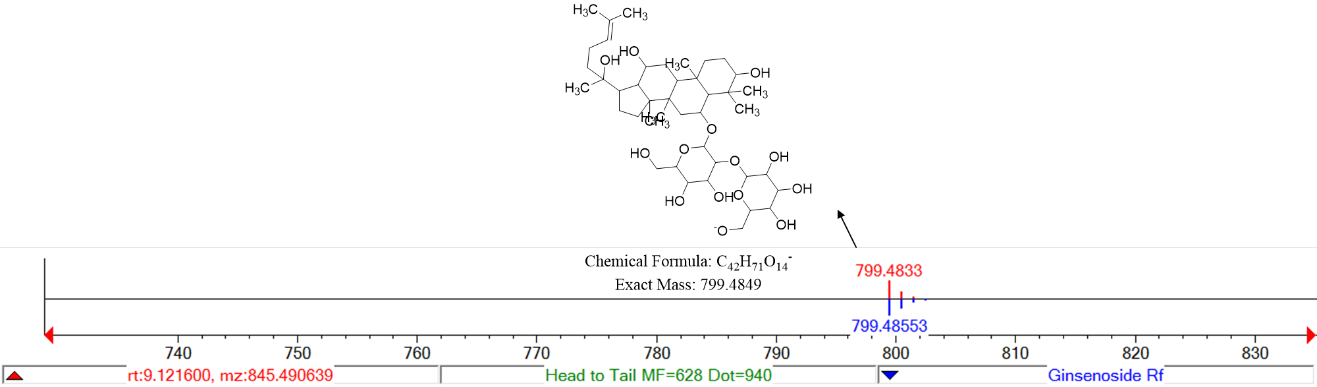


**11. Ginsenoside Rb1**


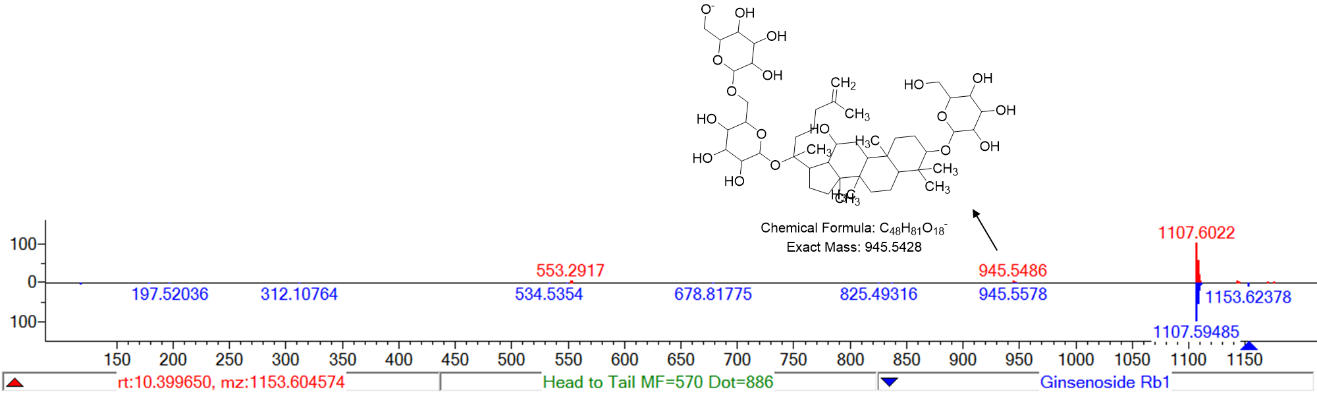


**12. Ginsenoside Rg3**


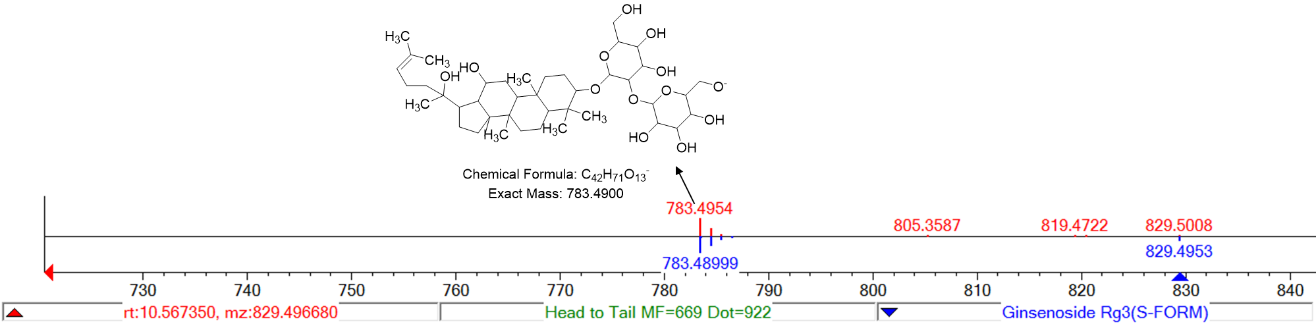


**Figure S2.** EIC chromatogram and MS^2^ spectra of each compound in ESI (-).


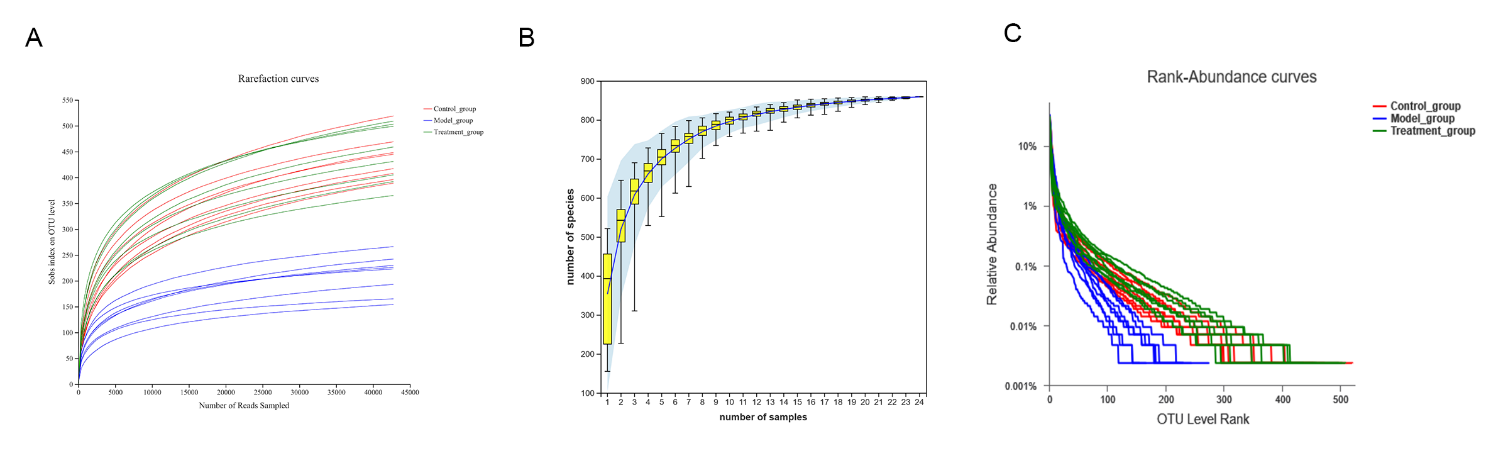
**Fig. S3.** Description and evaluation of sequencing data. (A) Rarefraction curves were performed to evaluate the sequencing data volume is adequate to cover nearly all of microorganisms. (B) Sample size was evaluated by species accumulation curves. (C) Richness and evenness of species was evaluated by rank abundance curve.


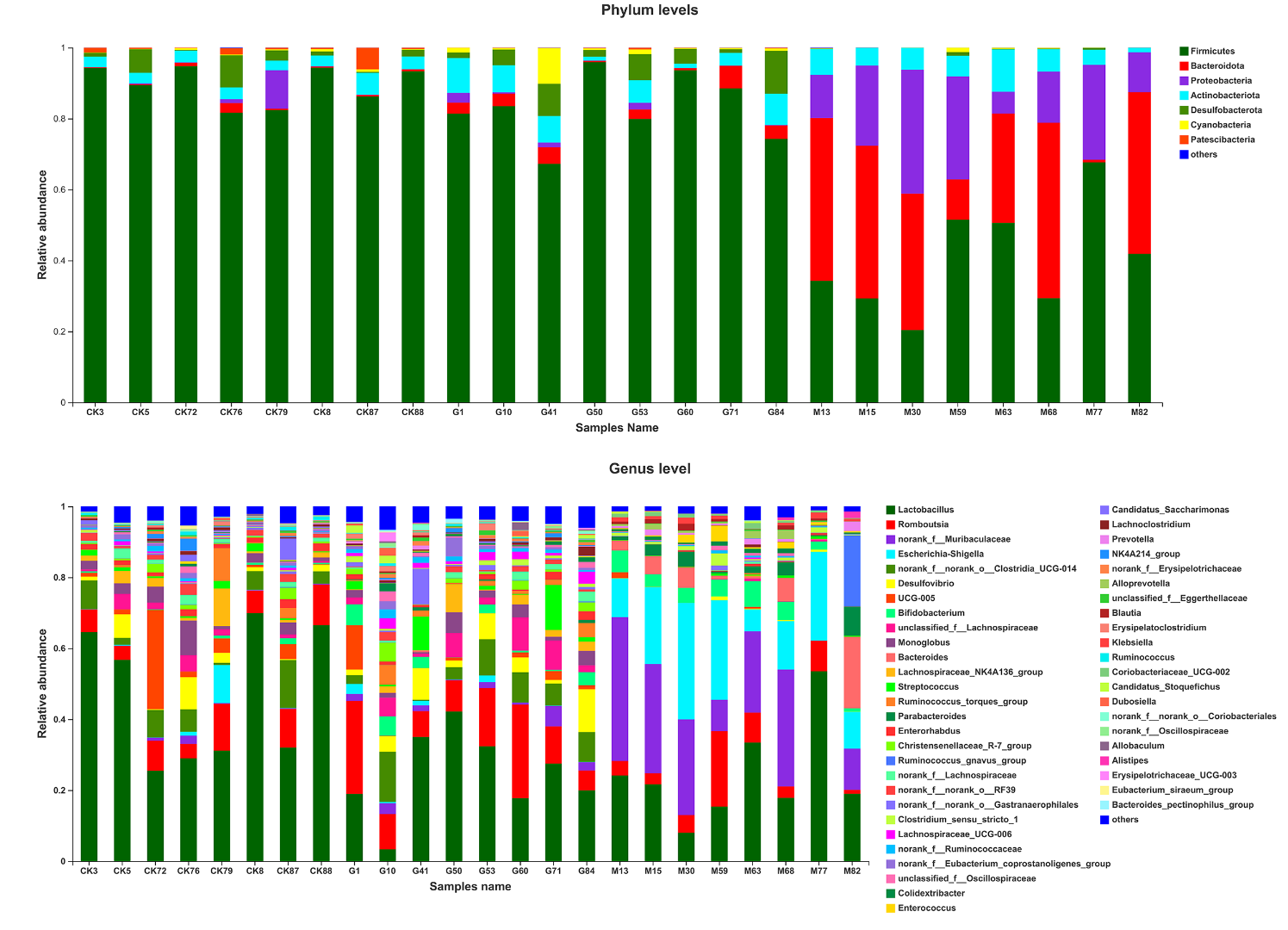


**Fig. S4.** Gut microbiota abundance of each sample at phylum level and genus level .


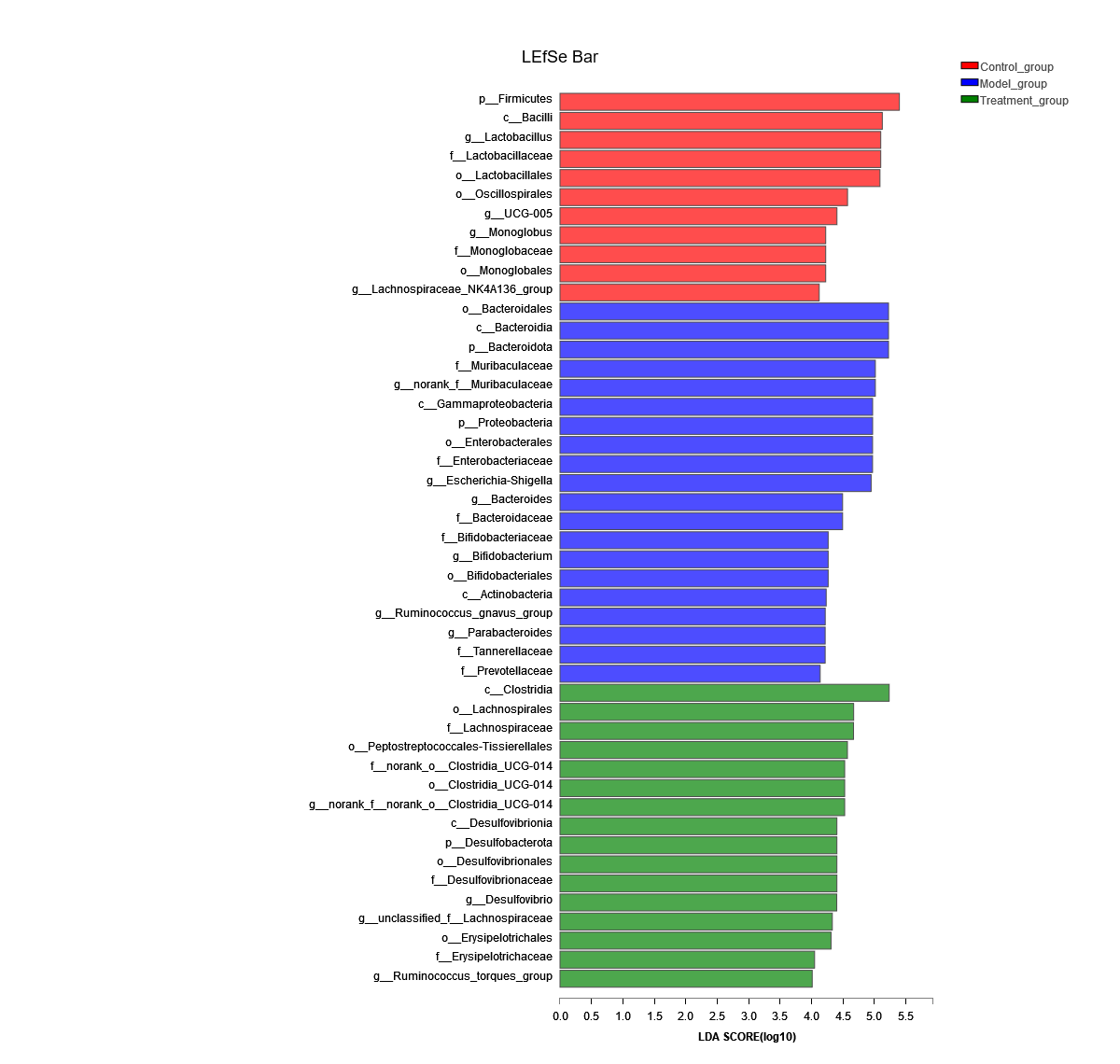


**Fig. S5.** (A) LEfSe analysis of relative abundance of gut microbiome among Control, Model and YXJYD-treatment groups. The larger of the LDA score represents the stronger effect of the species abundance on the differentiation. The Cut-off values were p < 0.05 and LDA > 4.


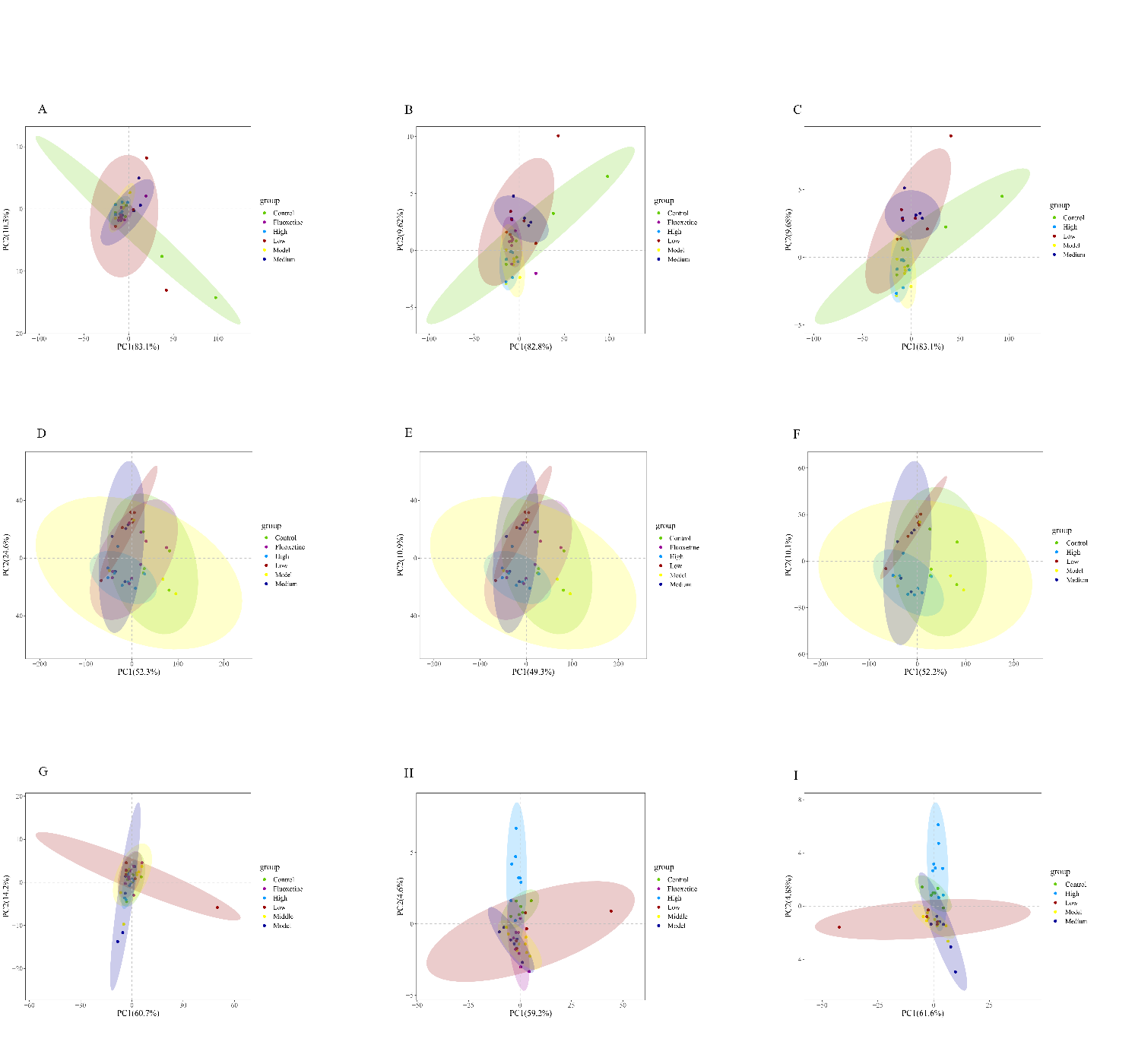


**Fig. 6.** Score plots of ^1^H NMR data from feces, urine, and serum samples. A, B, C. Score plot of fecal PCA analysis, PLS-DA analysis, and PLS-DA analysis(except Fluoxetine), respectively; D, E, F. Score plot of fecal PCA analysis, PLS-DA analysis, and PLS-DA analysis(except Fluoxetine), respectively; GHI, Score plot of fecal PCA analysis, PLS-DA analysis, and PLS-DA analysis(except Fluoxetine), respectively.


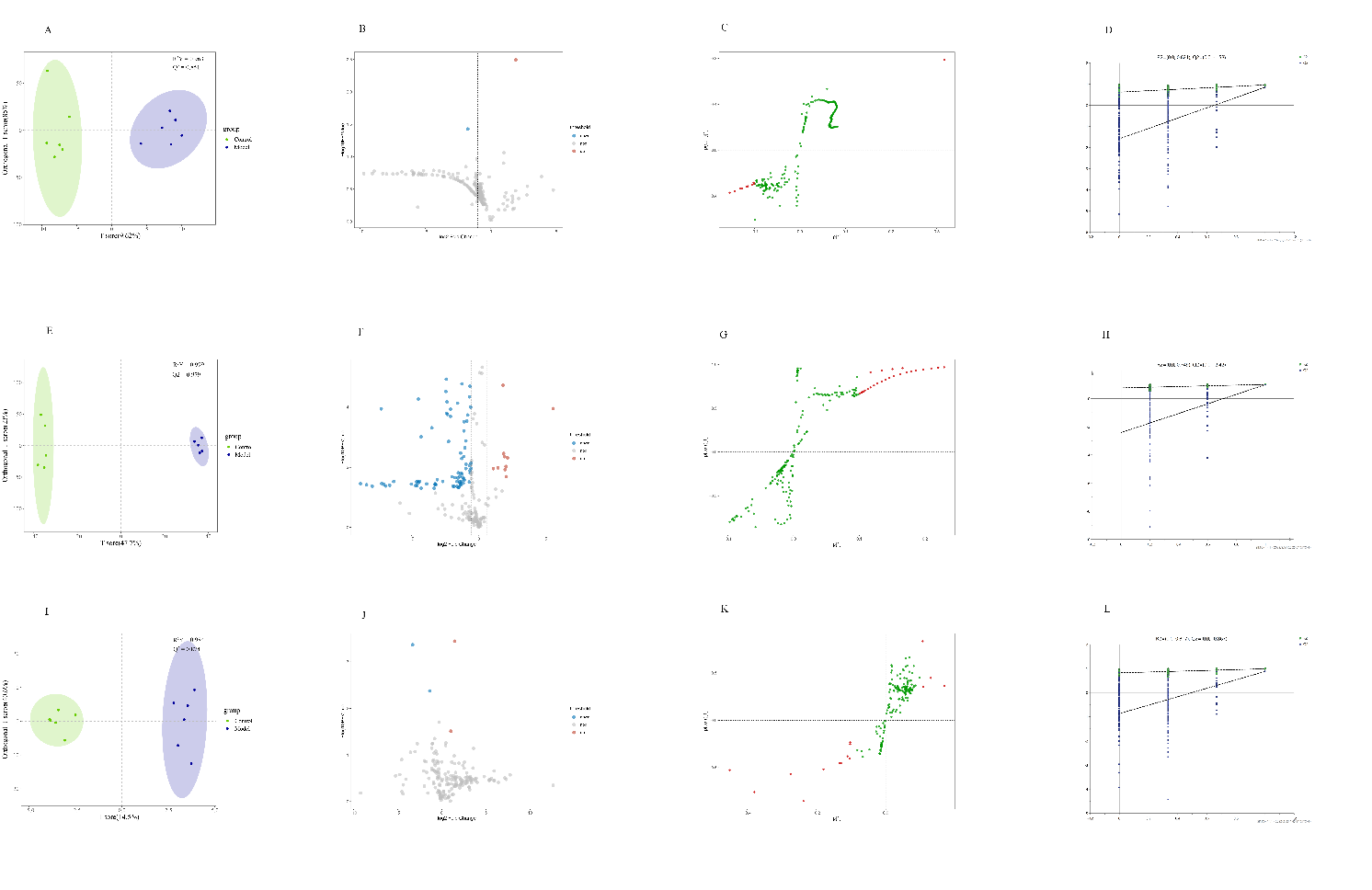


**Fig. S7.** Visible results of OPLS-DA. AEI, OPLS-DA scores plot of fecal, urine, and serum; BFJ, volcano plot of fecal, urine, and serum; CGK, S-plot of fecal, urine, and serum; DHL, result of 200-time permutation tests, fecal, urine, and serum.
